# Application of the thrombin generation assay in patients with antiphospholipid syndrome: A systematic review of the literature

**DOI:** 10.3389/fcvm.2023.1075121

**Published:** 2023-03-28

**Authors:** Rachel Gehlen, Arne Vandevelde, Bas de Laat, Katrien M. J. Devreese

**Affiliations:** ^1^ Department of Functional Coagulation, Synapse Research Institute, Maastricht, Netherlands; ^2^ Department of Biochemistry, Cardiovascular Research Institute Maastricht, Maastricht University Medical Centre, Maastricht, Netherlands; ^3^ Coagulation Laboratory, Ghent University Hospital, Ghent, Belgium; ^4^ Department of Diagnostic Sciences, Ghent University, Ghent, Belgium

**Keywords:** anitphospholipid antibodies, antiphospholid syndrome, pregnancy outcome, thrombin generation, thrombosis

## Abstract

**Background:**

The antiphospholipid syndrome (APS) is classified by the presence of antiphospholipid antibodies (aPL) and thrombotic and/or adverse obstetric outcomes. The diagnosis and risk assessment of APS is challenging. This systematic review investigated if the thrombin generation (TG) assay could be helpful for APS diagnosis and risk assessment.

**Methods:**

A systemic review was performed by searching two databases (MEDLINE and Embase) until March 31, 2022, using a search strategy with two concepts: APS and TG, and related keywords. Two reviewers independently screened the articles based on predefined inclusion and exclusion criteria. Data extraction and quality assessment with the Newcastle-Ottawa Scale (NOS) were performed independently. Synthesis Without Meta-analysis guidelines were followed for data synthesis reporting.

**Results:**

Fourteen studies with 677 APS and 1,349 control subjects were included with variable quality according to the NOS. Twelve studies measured TG *via* the calibrated automated thrombogram (CAT) method using a fluorogenic substrate, whereas two used a chromogenic substrate-based TG assay. One study compared the CAT assay to the fully-automated ST Genesia® (Stago, France). Two studies initiated TG using platelet-rich plasma, whereas the rest of the studies used platelet-poor plasma. Resistance to activated protein C (aPC) was examined in ten studies. They reported a significant increase in aPC-resistance in APS patients compared to healthy controls, aPL-carriers, and thrombotic controls. Based on two studies, the prevalence of aPC-resistance was higher in APS patients compared to healthy controls and thrombotic controls with odds ratios of 5.9 and 6.8–12.8, respectively (*p* < 0.05). In contrast, no significant difference in aPC-resistance was found between APS patients and autoimmune disease controls. Furthermore, 7/14 studies reported TG-parameters including peak height, endogenous thrombin potential, lag time, and time to peak, but these outcomes were highly variable between studies. Furthermore, TG methodology between studies differed greatly, impacting the comparability of the studies.

**Conclusion:**

aPC-resistance measured with TG was increased in APS patients compared to healthy and thrombotic controls, but the diagnostic and prognostic value is unclear compared to current diagnostic strategies. Studies of other TG-parameters were heterogeneous and more research is needed to identify their potential added value in APS diagnosis.

**Systematic Review Registration:**

https://www.PROSPERO/, identifier: CRD42022308363

## Introduction

The antiphospholipid syndrome (APS) is a systemic autoimmune disorder characterised by the persistent presence of antiphospholipid antibodies (aPL) and clinical features of thrombosis and/or pregnancy morbidity (1). aPL are a heterogeneous population of circulating immunoglobulins including anticardiolipin antibodies (aCL), anti-*β*2-glycoprotein I antibodies (a*β*2GPI), and lupus anticoagulant (LA) (1). The clinical phenotype associated with APS is highly heterogeneous, and pathophysiological mechanisms are still not fully understood. Despite the progress in unravelling the pathogenesis of APS (2), difficulties remain in identifying patients at high thrombotic and obstetric risk.

The diagnosis of APS relies predominantly on laboratory testing of aPL due to the relatively high incidence of clinical manifestations such as thrombosis and obstetric complications in the general population. Therefore, the laboratory detection of aPL is required to confirm the clinical suspicion of APS. The classic tests for the detection of aPL comprise clotting-based assays for the detection of LA and solid phase immunoassays for the detection of aCL and a*β*2GPI IgG/IgM antibodies (3). Although various studies have established an association between these laboratory assays and the clinical manifestations of APS (4–7), none of these assays is considered as the gold standard in APS. Consequently, several other laboratory tests and diagnostic tools are being investigated that may help to improve the diagnosis and risk evaluation in patients with APS.

Recently, interests have focused on more global coagulation assays such as the thrombin generation (TG) assay. The TG assay measures both thrombin formation and inhibition, which represents a significant part of the overall coagulation process. Because of this, TG reflects more closely what occurs *in vivo* compared to clotting-based assays by measuring the dynamic processes of thrombin generation (8–10). Furthermore, TG tests can be used to study the contribution of procoagulants and anticoagulants in patients with various haemostatic disorders (11, 12), or to investigate the impact of medication on haemostasis (13, 14). TG methods lack standardisation (15), but while efforts are ongoing to improve this issue, the TG assay appears to be a valuable tool for determining the increased tendency to form blood clots (hypercoagulability) in patients with a wide variety of thrombotic disorders (11, 14). In addition, TG assays can also be used to assess activated protein C (aPC) resistance (aPC-r), a possible contributor to hypercoagulability in APS patients (16).

This systematic review aimed to investigate if the TG assay could be used as a diagnostic tool for, on one hand, the diagnosis of patients with APS and, on the other hand, for identifying APS patients at high risk for recurrent clinical manifestations.

## Methods

### Protocol and registration

This systematic review was registered in the International Prospective Register of Systematic Reviews (PROSPERO), registration number CRD42022308363. The review was reported according to the Preferred Reporting Items for Systematic Reviews and Meta-analyses (PRISMA) 2020 guidelines (17).

### Search strategy

Two databases (MEDLINE *via* the PubMed interface and Embase *via* the Embase.com interface) were searched for the combination of two concepts (APS and TG) according to the search strategy specified in Supplementary Figure S1. No restrictions such as language or date specification were applied. Validation of the search strategy was performed by adequate identification of a validation set of six studies specified by K.D. (Supplementary Table S1). The search was performed on November 3, 2021. An e-mail alert was activated on both databases to receive weekly updates and these studies were included for evaluation until March 31, 2022.

### Eligibility and selection process

Duplicate removal was performed in EndNote 20 (Clarivate, London, United Kingdom) with manual verification. Deduplicated records were transferred to Rayyan (Rayyan Systems Inc., Cambridge, MA, United States) for the screening process (18). Two researchers (R.G. and A.V.) independently reviewed all records for eligibility based on title and abstract. After the screening stage, the same reviewers independently evaluated the full texts of the included articles. In case of disagreement, a third reviewer (K.D.) was consulted to reach a consensus at both evaluation stages. Eligibility was checked against predefined criteria (Table 1). Percentage agreement and Cohen's kappa values were determined at both stages.

**Table 1:** Eligibility criteria for assessing inclusion and exclusion of retrieved research.

Variable	Inclusion	Exclusion
Population	– Adult patients diagnosed with the antiphospholipid syndrome or who met the Sapporo/Sydney classification criteria.	– Underaged patients (<18 years). – No exclusion based on sample size.
Exposure	– Assessment of thrombin generation with a thrombin generation assay.	– No exclusion based on type of thrombin generation assay used.
Control	– Laboratory assays: antiphospholipid antibodies included in classification criteria. – Clinical: patients without antiphospholipid syndrome.	– Studies describing only laboratory parameters not included in classification criteria (e.g. anti-phosphatidylserine/prothrombin antibodies).
Outcomes	– Description of thrombin generation in relation to classification of antiphospholipid syndrome or related clinical risk (thrombosis or pregnancy morbidity).	– Studies describing only clinical manifestations not included in classification criteria (e.g. thrombocytopenia). – No thrombin generation results or derived parameters reported.
Study design	– Observational studies including case-control, cohort, and cross-sectional studies.	– Article type: letter, conference abstract, (systematic) review, editorials. – Non-human studies. – Experiments based on patient-derived material such as isolated antibodies. – Language other than English.

### Data extraction and quality assessment

Data extraction and quality assessment were performed independently by two reviewers (R.G. and A.V.). Extracted data were registered in a table agreed upon beforehand in Excel (Microsoft, Redmond, WA, United States.). Data items that were extracted are summarised in Supplementary Table S2. A third reviewer (K.D.) was consulted in case of disagreement when discussion between the two reviewers did not lead to consensus. The Newcastle-Ottawa Scale (NOS) was applied to assess the quality of the included cohort and case-control studies (19). An adapted NOS was used for scoring studies with a cross-sectional design (Supplementary Figure S2) (20). In the (adapted) NOS, each study was attributed stars for three main items (selection, comparability, and outcome/exposure). The number of stars was added up per study, resulting in a score of maximally nine stars for case-control studies and seven stars for cross-sectional studies. The higher the score, the higher the expected quality and the lower the risk for bias in the study. High-quality case-control studies are mostly considered with a NOS score of seven or more, while no generally accepted threshold is available for cross-sectional studies. Both reviewers independently provided a score for each included article, and it was reported after a consensus was reached between the two reviewers.

### Data synthesis

No meta-analysis was performed owing to the incomplete reporting of effect estimates and diversity of exposures (TG methodologies), control groups and study designs. Synthesis Without Meta-analysis (SWiM) guidelines were followed for data synthesis reporting (21). Studies were grouped based on the outcome reported, being aPC-r and other TG-derived parameters, because of their different underlying concept. Studies may however report both outcomes and can therefore be included in both groups. No standardised metric could be used to describe continuous data from aPC-r results because of the variety in TG methods used to assess aPC-r. It was not possible to combine *p*-values because exact values were not always available, but the direction of the effect was synthesised. Unadjusted odds ratios (ORs) were calculated without pooling if a clinical cut-off value was available. Data were presented per group in a tabular format (separately for continuous and dichotomous data) and ungrouped graphically in a modified effect direction plot with studies ordered according to the score received during the quality assessment, followed by study patient group size and split by study design (22). No formal heterogeneity assessment was performed.

## Results

### Publication characteristics

**Figure 1:**
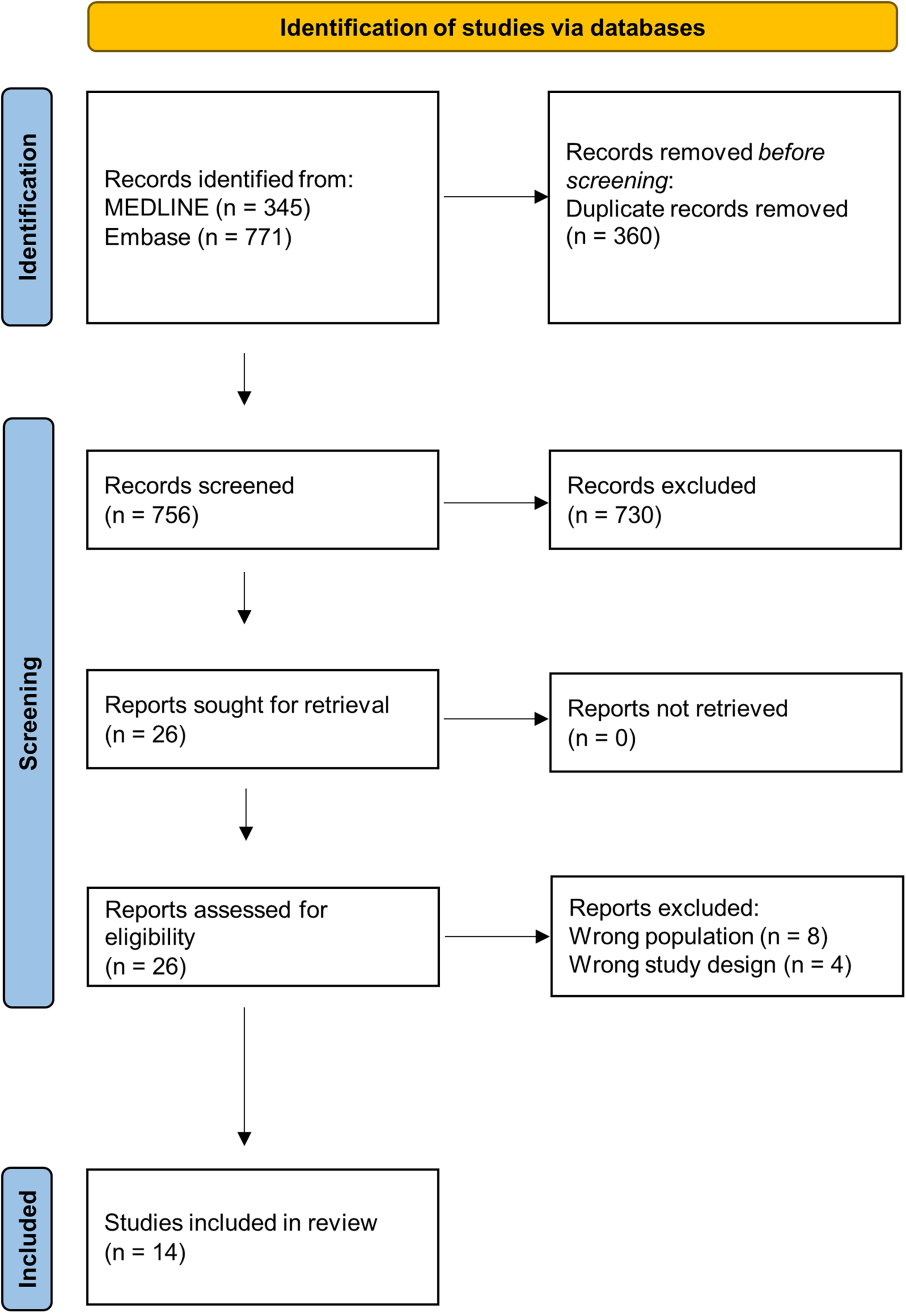
Flow chart of the systematic search of the literature and study selection process.

In total, 1,116 records were retrieved from the two databases. After removing duplicates, 756 records were screened for eligibility, of which 26 were selected for full-text evaluation. After full-text evaluation, 14 records were included for data extraction (Figure 1). The agreement between the two reviewers for the abstract and full-text screening was substantial with values of 99% (Cohen's *κ*: 0.72) and 81% (Cohen's *κ*: 0.61), respectively. Reasons for exclusion of the articles that were subject to full-text evaluation can be found in Supplementary Table S3 (23–34). Only two of the 14 included studies were performed outside of Europe (one in Japan and one in Canada). We identified multiple records from the same research groups. We could rule out overlapping populations between studies for some research groups, although for three studies overlap is not unlikely since they use patient populations recruited under the same ethical reference number (35–37). However, the information provided in these three studies is relevant and unique since they compare the APS patient population against distinct control groups. All studies were retrospective and had an observational design, of which four were identified as cross-sectional studies, ten case-control studies, and zero cohort studies. Characteristics and main findings of all included studies are summarised in Supplementary Table S4 (35–48). Only one record described information on the ethnicity of the cases (37). Therefore, information on ethnic characteristics was not further reported.

### Quality assessment

The quality assessment based on the NOS is shown for each study in Supplementary Table S5. Only one study reached a NOS score of 7 and could be considered of high quality. All other case-control studies had a score of 5–6 except for one study that scored 3 stars. Three cross-sectional studies had a score of 4, and one study scored 3 stars. One study received points in the comparability category since no other studies controlled for confounders such as age or gender.

### Patient and control characteristics

Six hundred and seventy-seven APS cases were reported. APS can be classified as thrombotic and obstetric APS (1). Most studies identified thrombotic and obstetric manifestations retrospectively based on medical records. However, only eight studies specified the location or type of thrombotic and obstetric adverse events (35, 37, 38, 43, 45, 46, 36, 44). Two studies distinguished thrombotic and obstetric APS patients as separate patient groups (36, 38), including 118 thrombotic APS patients and 34 obstetric APS patients (36, 38). Four studies included thrombotic APS patients (*n* = 102) only (35, 37, 43, 47). Four studies specified the proportion of APS patients with thrombotic manifestations (*n* = 200), obstetric manifestations (*n* = 37), or both (*n* = 14) but did not analyse the subgroups separately (40, 41, 45, 46). In four studies, no clear differentiation between thrombotic and obstetric APS patients could be derived from the publications (*n* = 172) (39, 42, 44, 48). In addition, APS patients can be classified according to the presence (i.e., secondary APS) or absence (i.e., primary APS) of any coexisting autoimmune disease (AID) (49). In one study, only secondary APS patients (*n* = 24) were included (37). Three studies specified whether patients had primary (*n* = 168) or secondary (*n* = 39) APS and interpreted the subgroups separately (36, 44, 48). The presence of AID was not specified in 212 APS patients (39, 41–43, 46, 47). Furthermore, in the remaining studies 42 secondary APS and 192 primary APS patients were investigated, but no subgroup analysis was performed in these studies (35, 38, 40, 45).

In total, 1,349 control cases were reported. Control cases can be categorised into four groups according to the clinical presentation reported. Group 1 included 127 cases that were persistently positive for aPL but did not fulfil the clinical criteria manifestations of APS. Group 2 included 207 thrombotic control patients with a history of thromboembolic complications but did not fulfil the APS laboratory criteria (3). Group 3 included 214 AID patients with conditions other than APS. Most of these patients had systemic lupus erythematosus (SLE) (*n* = 179), either isolated SLE (67/179), with the presence of aPL (69/179), or with a history of thrombotic complications (14/179), but none of these patients fulfilled the APS criteria (3). Group 4 included 639 presumably healthy control cases. Three studies included patient groups that could not be described by the groups above (40, 45, 46). De Laat-Kremers et al. included 93 hospital controls, which were patients visiting the hospital for conditions other than APS (40). In addition, two studies reported anticoagulant-matched controls that did not meet the APS classification criteria (45, 46). Kremers et al. included 31 vitamin K antagonist (VKA) treated control cases treated for indications other than APS (45). Liestøl et al. included 38 patients on long-term warfarin therapy for non-valvular atrial fibrillation (46).

### Thrombin generation methodology

Preanalytical and analytical aspects of all included studies are summarised in Table 2. Twelve articles measured TG *via* the calibrated automated thrombogram (CAT) method, according to the principles described by Hemker et al. in 2003. One study compared the CAT assay to the ST Genesia® (Stago, Asnières-sur-Seine, France) methodology, an automated TG analyser based on the same principles as the CAT assay (36). TG methods can be performed using platelet-rich plasma (PRP), platelet-poor plasma (PPP), or whole blood. Zuily et al. and Foret et al. initiated TG in PRP (42, 48), whereas the rest of the studies used PPP. The platelets in the PRP provide a natural source of phospholipids. However, in PPP, negatively charged phospholipids need to be added to the reaction for optimal TG. In most publications, the use of synthetic phospholipids at a final concentration of 4.0 *μ*M was reported (35–38, 40, 45–47), although only two studies specified the phospholipid composition (45, 46). Devreese et al. lowered the phospholipid concentration to 1.0 *μ*M to raise the phospholipid dependency of the assay (41). Bloemen et al. used inverted erythrocyte membranes as a phospholipid surface (39). In all studies with CAT, TG was triggered by tissue factor (TF). The final TF concentration was 5.0 pM for the majority of the studies using the CAT method (35–37, 39–41, 45, 46). Two studies also included a lower TF concentration of 1.0 pM to make the TG measurement more sensitive to the intrinsic coagulation pathway (40, 45). Billoir et al. and Zuily et al. did not use 5.0 pM TF to trigger TG but used a lower TF concentration of 1.0 pM and 0.5 pM, respectively (38, 48). In addition, Matsumoto et al. initiated TG utilising a mixture of TF (0.5 pM) and ellagic acid (0.3 *μ*M) to investigate the thrombogram but used 1.0 pM TF to assess aPC-r. This reaction mixture can trigger thrombin generation *via* both intrinsic and extrinsic coagulation mechanisms (47). Finally, Foret et al. reported the use of a low TF concentration to initiate TG, but no exact TF concentration was reported (42).

**Table 2:** Preanalytical and analytical aspects of the included studies.

First author	Year	Preanalytical					Thrombin generation methodology									
		PPP/PRP	Sample preparation	Storage	Thawing	Mixing with PNP	CAT	Instrument	Software	Replicate amount	TF f.c.	TF origin	PL f.c.	PL origin	Nor-malised	Normalisation method
**Arachchillage**	2014	PPP	Citrated plasma, double centrifugation: 15 min., 2000 *g* at RT	−80°C	10 min. 37 °C WWB	Yes 1:1 (sample:PNP)	Yes	n.s.	n.s.	Triplicate	5.0 pM	Synthetic	4.0 µM	Synthetic	Yes	Against in-house prepared PNP in each run
**Billoir**	2021	PPP	n.s.	n.s.	n.s.	No	n.s.	Fluoroskan Ascent (Thermo Fisher Scientific, Finland)	n.s.	n.s.	1.0 pM	Synthetic	4.0 µM	Synthetic	No	Not applicable
**Bloemen**	2016	PPP	Citrated plasma, filtration (0.2 µm filter)	n.s.	n.s.	No	Yes	n.s.	Throm-binoscope (Nether-lands), version n.s.	n.s.	5.0 pM	Recom-binant, human (Dade Behring, Germany)	n.s.	Erythrocyte membranes	No	Not applicable
**De Laat – Kremers**	2021	PPP	Citrated plasma, double centrifugation: 10 min., 2821*g*	n.s.	n.s.	No	Yes	n.s.	n.s.	n.s.	1.0 pM 5.0 pM	Synthetic	4.0 µM	Synthetic	No	Not applicable
**Devreese**	2010	PPP	Citrated plasma, double centrifugation: 15 min., 2000*g* and 10 min.,>2500 *g* at RT	−80°C	n.s.	Yes 1:1 (sample:PNP)	Yes	n.s.	n.s.	Duplicate	5.0 pM	n.s.	1.0 µM	n.s.	Yes	Against in-house prepared PNP in each run
**Efthymiou**	2022	PPP	Citrated plasma, double centrifugation: 15 min., 2000 *g* at RT	−80°C	10 min. 37 °C WWB	Yes 1 : 1 (sample:PNP) if VKA therapy	1. Yes 2. No	1. n.s. 2. ST Genesia (Stago, France)	1. n.s. 2. ST Genesia	1. n.s. 2. Duplicate	1. 5.0 pM 2. n.s.	1. Synthetic 2. Recombinant human	1. 4.0 µM 2. n.s.	1. Synthetic 2. n.s.	1. n.s. 2. No	1. n.s. 2. Not applicable
**Foret**	2021	PRP	Citrated plasma, PRP: 10 min., 190 *g* at 20°C + addition of PPP (centrifugation: 10 min., 2000 *g* at 20°C) to obtain 150 G/L platelets	No storage (fresh PRP)	No storage	No	Yes	Fluoroskan Ascent (Thermo Fisher Scientific, Finland)	Throm-binoscope (Nether-lands), version n.s.	Triplicate	n.s.	Recom-binant, human (Dade Behring, Germany)	n.s.	PRP.	No	Not applicable
**Green**	2012	PPP	Citrated plasma, double centrifugation: 15 min., 2000*g* at RT	−80°C	37°C, n.s.	No	No	ACL TOP500 (Werfen, Spain)	n.s.	Duplicate	1.5 pM	Recom-binant human (Recom-biplastin 2 G) (Werfen, Spain)	10 µg/ mL	n.s.	Yes	Against commercial PNP (Techno-clone, Austria) in each run
**Hanly**	2000	PPP	Citrated plasma, single centrifugation: 30 min., 3000*g*	−70°C	n.s.	Yes 1:2 (sample: PNP*)	No	In house chromogenic thrombin generation assay	n.s.	Triplicate	n.s.	Thrombo-plastin (Sigma, US)	n.s.	Thrombo-plastin (Sigma, US)	No	Not applicable
**Kremers**	2018	PPP	Citrated plasma, double centrifugation: 10 min., 2821 *g*	−80°C	n.s.	No	Yes	n.s.	Throm-binoscope (Nether-lands), version n.s.	n.s.	1.0 pM 5.0 pM	Recom-binant n.s. (Stago, France)	4.0 µM	Synthetic: 60 mol% DOPC, 20 mol% DOPS, and 20 mol% DOPE (n.s.)	No	Not applicable
**Liestøl**	2007	PPP	Citrated plasma: centrifugation 15 min., 2000g at 20 °C + filtration [Millex-GV 0,22 µm Filter Unite, Millipore, France]	−70°C; part of samples were transported at RT for 1-2 days between centrifu-gation and filtration	n.s.	Yes 1:1 (sample:PNP)	Yes	Fluoroskan Ascent (Thermo Fisher Scientific, Finland)	Throm-binoscope (Nether-lands), version n.s.	n.s.	5.0 pM	Recom-binant, human (Dade Behring, Germany)	4.0 µM	Synthetic: 60 mol% PC, 20 mol% PS, and 20 mol% PE (Avanti Polar Lipids, US)	Yes	Against in-house prepared PNP in each run
**Matsumoto**	2017	PPP	Citrated plasma, single centrifugation: 15 min., 1500 *g*	-80°C	37°C, n.s.	No	Yes	Fluoroskan Ascent (Thermo Fisher Scientific, Finland)	Throm-binoscope (Nether-lands), version n.s.	n.s.	0.5 pM 1.0 pM	Recom-binant, human (Dade Behring, Germany)	4.0 µM	n.s.	Yes	Against in-house prepared PNP in each run
**Ramirez**	2021	PPP	Citrated plasma, double centrifugation: 15 min., 2000*g* at RT	-80°C	10 min. 37 °C WWB	Yes 1:1 (sample:PNP) if VKA therapy	Yes	n.s.	Throm-binoscope (Nether-lands), version n.s.	n.s.	5.0 pM	Synthetic	4.0 pM	Synthetic	No	Not applicable
**Zuily**	2012	PRP	Citrated plasma, PRP: 10 min., 190 *g* at 20°C + addition of PPP (centrifugation: 10 min., 1750*g*) to obtain 150 × 10^9^ platelets/L if platelet count in PRP >150 × 10^9^/L	No storage (fresh PRP)	No storage	No	Yes	Fluoroskan Ascent (Thermo Fisher Scientific, Finland)	Throm-binoscope (Nether-lands), version n.s.	n.s.	0.5 pM	Recom-binant, human (Dade Behring, Germany)	n.s.	PRP	No	Not applicable

*PNP, but not platelet poor Abbreviations: CAT, calibrated automated thrombogram; DOPC, dioleoyl phosphatidylcholine; DOPE, dioleoyl phosphatidylethanolamine; DOPS, dioleoyl phosphatidylserine; f.c., final concentration; n.s., not specified; PC, phosphatidylcholine; PE, phosphatidylethanolamine; PL, phospholipids; PNP, pooled normal plasma; PPP, platelet-poor plasma; PRP, platelet-rich plasma; PS, phosphatidylserine; RT, room temperature; TF, tissue factor; US, United States; VKA, vitamin K antagonist; WWB, warm water bath.

Furthermore, Green et al. and Hanly et al. reported a TG method different from CAT, using a chromogenic substrate for thrombin instead of a fluorogenic substrate used in CAT (43, 44). Green et al. used thromboplastin (Recombiplastin 2 G, Werfen, Spain) as TF source with a final concentration of 1.5 pM TF and 10 *μ*g/ml unspecified phospholipids to trigger thrombin generation in PPP (43). Hanly et al. also used thromboplastin (Sigma, St. Louis, MO, United States), in a final dilution of 1/80 as trigger reagent (44).

Resistance to aPC may be assessed by measuring and comparing TG with and without adding a protein C pathway activator (or aPC itself) to the sample. aPC-r was assessed in 71% (10/14) of all included studies (35–38, 42, 43, 45–48). In these studies, aPC-r was determined by adding aPC (5/10 studies) (38, 42, 46–48), or a protein C activator isolated from snake venom, Protac® (Pentapharm AG, Switzerland) (1/10 studies) (43) to a TG assay. Two studies used both Protac® and aPC to determine aPC-r (35, 37). One study used recombinant human thrombomodulin (TM) (Asahi Kasei Pharma, Beijing, China) to mediate protein C activation (45). In another study, aPC-r was determined using the CAT TG method by adding aPC and Protac® and using the TG analyser ST-Genesia® that uses TM from purified rabbit lung (36).

Anticoagulants can influence TG results. The use of anticoagulation therapy was reported in eight out of the 14 articles (35–37, 40–42, 45, 46). Devreese et al. and Liestøl et al. equally included patients with and without oral anticoagulant therapy. They reported that 18% (INR: 0.97–2.44) and 65% (INR: 1.6–4.3) of the total study population were on VKA treatment, respectively (41, 46). In both studies patient plasma was mixed with equal volumes of pooled normal plasma (PNP) to correct for reduced coagulation factor activity as a result of VKA treatment (41, 46). Ramirez et al. specified that 79.2% of the included APS patients were on anticoagulant therapy at the time of blood sampling (37). Of these patients, 1/19 was on low molecular weight heparin (LMWH), 4/19 on direct oral anticoagulants (DOACs), and 14/19 on VKA. To neutralise the effects of this anticoagulant therapy they reported mixing of patient plasma with equal volumes of PNP (37). In addition, Kremers et al. investigated the effect of VKA treatment on TG by including VKA-treated APS patients (*n* = 50), APS patients without VKA treatments (*n* = 30), and age- and gender-matched VKA-treated control subjects (*n* = 31) (45). De Laat-Kremers et al. described a validation cohort of APS patients and specified that 82% of these patients were on anticoagulants without describing further details (40). Foret et al. reported that 1.7% of the total population was on LMWH, whereas 45.3% was on VKA treatment (42). However, these percentages also include control patients, and details regarding anticoagulant use in the APS patient population alone were not provided (42). Two studies only included APS patients and thrombotic controls on VKA treatment (35, 36). Both studies corrected for possible effects of anticoagulant therapy by mixing 1:1 with PNP. Bloemen et al. included three VKA-treated patients and two patients without anticoagulants to determine the effect of anticoagulant on TG (39). Three studies only included patients without anticoagulants or patients that had stopped treatment (38, 43, 48). In addition, two studies did not provide details regarding anticoagulant use in APS patients (44, 47).

### Outcomes

Multiple parameters can be investigated when using a TG assay. Resistance to aPC is considered when there is less inhibition of TG than expected when activating the protein C pathway. Eight studies reported continuous data of results on which aPC-r assessment was based. They reported the average or median values with standard deviation (SD), 95% confidence interval (95% CI) or interquartile range (IQR) (Table 3). Three studies reported and compared the positivity rate or prevalence of aPC-r in APS and control subjects (Table 4) (36, 37, 43). ORs were calculated based on the available data for two studies (36, 43). aPC-r was assessed based on normalised inhibition of endogenous thrombin potential (ETP) (35, 46) or peak height (PH) (47), non-normalised inhibition of ETP (36, 37) or PH (45), ETP ratio (38, 42), normalised area under the TG curve (AUC) ratio (43), and aPC concentration needed to obtain 50% ETP inhibition (IC50-aPC) (48). Foret et al. found a significantly higher aPC-r in patients with APS compared to patients with the presence of aPL without clinical APS manifestations (*p* = 0.04), but not compared to a group of isolated AID patients without the presence of aPL (42). Arachchillage et al. observed a higher aPC-r, expressed as a lower normalised ETP inhibition with aPC and Protac®, in VKA-treated thrombotic APS patients compared to a VKA-treated control group and a healthy control group (35). Interestingly, they also observed a significantly different aPC-r between both control groups when using Protac®, but not if aPC was used (35). Efthymiou et al. demonstrated a significant association between aPC-r and primary APS patients compared to thrombotic controls when using TM, aPC, or Protac® with OR ranging from 6.8 to 12.8 (36). In contrast, no significant difference was observed in APS patients compared to SLE patients without thrombotic history (*p* > 0.05) (36). Similarly, Ramirez et al. could not demonstrate a significant difference in the prevalence of aPC-r between a group of secondary APS patients and SLE patients without a thrombotic history including both aPC and Protac® methods for aPC-r determination (37). Efthymiou et al. also compared the use of TM, aPC, and Protac® for determining aPC-r which showed poor agreement, but a comparable positivity rate based on in-house determined cut-off values (36). Kremers et al. showed that APS patients had significantly higher aPC-r compared to VKA treatment-matched controls, demonstrated by a lower ETP inhibition after adding TM (45). Liestøl et al. also demonstrated a higher level of aPC-r in VKA-treated and untreated APS patients compared to VKA-treated and untreated controls, respectively (46). Considering the overall APS group (*n* = 52), the median normalised ETP inhibition was significantly lower compared to a group of LA-positive patients without APS (44%, 95% CI: 30.1–55.7 vs. 78.8%, 95% CI: 73.9–95.8), corresponding to a higher aPC-r in APS patients (46). Similarly, Matsumoto et al. demonstrated a higher aPC-r in the LA-positive APS group compared to LA-positive controls, but with high variation within the groups (32). Zuily et al. showed that the IC50-aPC was higher in primary and secondary APS patients compared to presumably healthy controls (48). Although, non-APS SLE patients also showed a higher IC50-aPC (48). Billoir et al. observed that both thrombotic and obstetric APS patients had significantly higher aPC-r compared to healthy controls and aPL-carriers (*p *= <0.001) (38). This was also confirmed by Green et al. who demonstrated a higher rate of aPC-r in thrombotic APS patients compared to healthy controls (OR 5.9, *p* = 0.005), using a chromogenic substrate-based TG method (43).

**Table 3:** Summary of continuous outcomes of thrombin generation based resistance to activated protein C.

**First author (year)**	**aPC-r method**	**Patient group (*n*)**	**aPC-r results patients**	**Control group (*n*)**	**aPC-r results controls**	**p-value**	**Remarks**
**A. Cross-sectional studies**							
Foret (2021)	Ratio of ETP (CAT) with aPC and without aPC using PRP**	Primary and secondary APS (82)	0.583 +/- 0.278	1. AID controls (15)	1: not specified	>0.05	None
				2. aPL-carriers (20)	2: 0.423 +/- 0.239	0.04	
Arachchillage (2014)	Normalised inhibition (%) of ETP (CAT) with Protac and rhAPC using PPP 1:1 mixed with PNP*	VKA-treated TAPS (51)	Protac: 66.0% (59.5-72.6) rhAPC: 81.3% (75.2-88.3)	1. VKA-treated thrombotic controls (51)	1. Protac: 80.7% (74.2-87.2)	0.007	Comparing the two control groups: aPC-r with Protac significantly different between both control groups.
					1. rhAPC: 97.7% (93.6-102)	0.002	
				2. Healthy controls (51)	2. Protac: 102% (96.2-108)	<0.0001	
					2. rhAPC: 98.3% (92.2-104)	0.01	
**B. Case-control studies**							
Kremers (2018)	Median inhibition (%) of PH (CAT) with TM using PPP	A. VKA-treated APS (50)	A: 15%	1. VKA-treated controls (31)	1: 35%	A-1: <0.001	None
		B. APS without VKA (30)	B: 10%	2. Healthy controls (45)	2: 50%	B-2: <0.001	
Liestøl (2007)	Normalised inhibition (%) of ETP (CAT) with aPC using PPP 1:1 mixed with PNP*	A. VKA-treated APS (34)	A: 33.8% (28.8-55.7)	1. VKA-treated controls (38)	1: 115% (111-122)	A-1: <0.001	Overall patient group had median ETP inhibition of 44.6% (95%CI: 30.1-55.7) and was significantly different from control group 2 with LA presence (p=0.003).
				2. LA-positive controls (29)	2: 78.8% (73.9-95.8)	Not available	
		B. APS without VKA (18)	B: 52.0% (41.0-81.2)	3. Healthy controls (53)	3: 107% (106-108)	B-3: <0.001	
Zuily (2012)	APC concentration (nM) necessary for 50% ETP inhibition (CAT) after addition of aPC in PRP (=IC50-aPC) ***	Not applicable	Not applicable	1. SLE controls without aPL (13)	1: 27.3 nM (23.5-43.5)	Not available	IC50-aPC of SLE controls without aPL presence was also significantly increased compared to controls.
		A. Primary APS (38)	A: 15.3 nM (9.7-34.0)	2. Controls, not specified (39)	2: 10.4 nM (8.5-15.8)	A-2: <0.05	
		B. Secondary APS (10)	B: 64.1 nM (25.9-65.0)			B-2: <0.05	
Billoir (2021)	Ratio (%) of ETP (CAT) with aPC and without aPC using PPP**	A. TAPS (19)	A: 52% +/- 16.4	1. aPL-carriers (11)	1: not specified	A-1: <0.001	Significant difference between patient groups and aPL-carriers, but values not mentioned.
						B-1: not specified	
		B. OAPS (11)	B: 64.1% +/- 14.6	2. Healthy controls (25)	2: 27% +/- 13.8	A-2: <0.001	
						B-2: <0.001	
Green (2012)	Normalised ratio of AUC from TG assay with and without Protac in PPP***	TAPS without anticoagulation (17)	1.1 (0.8-1.4)	Healthy controls (35)	2.8 (2.4-4.7)	<0.001	Other thrombotic patients with protein C/S deficiency or FV Leiden had also significantly different normalised AUC ratio compared to healthy controls.
Matsumoto (2016)	Normalised inhibition (%) of PH (CAT) with aPC using PPP**	APS, LA-positive (10)	5% +/- 7	1. LA-positive controls (10)	1. 42% +/- 39	1. <0.01	The normalised inhibition of PH by aPC was also significantly lower in LA-positive controls compared to healthy controls.
				2. Healthy controls (not specified)	2. Not specified	2. <0.01	

^*^ Median (95% CI);

^**^ Mean +/- SD;

^***^ Median (IQR)

Abbreviations: AID, autoimmune disease; aPC, activated protein C; aPC-r, activated protein C resistance; aPL, antiphospholipid antibodies; APS, antiphospholipid syndrome; AUC, area under the thrombin generation curve; CAT, calibrated automated thrombography; ETP, endogenous thrombin potential; FV, Factor V; LA, lupus anticoagulant; OAPS, obstetric APS; PH, peak height; PNP, pooled normal plasma; PPP, platelet-poor plasma; PRP, platelet-rich plasma; rhAPC, recombinant human activated protein C; SLE, systemic lupus erythematosus; TAPS, thrombotic APS; TG, thrombin generation; TM, thrombomodulin; VKA, vitamin K antagonist; VTE, venous thromboembolism.

**Table 4:** Prevalence and positivity rate of thrombin generation-based resistance to activated protein C (dichotomous outcome).

**First author (year)**	**aPC-r method**	**Cut-off value**	**Patient group (*n*)**	**Patients with aPC-r**	**Control group (*n*)**	**Controls with aPC-r**	**Odds ratio** **(95% CI)**	**p-value**
**A. Cross-sectional studies**								
Efthymiou (2022)	Inhibition (%) of ETP on PPP with: A. Protac (CAT); B. rhAPC (CAT); C. TM (ST Genesia). Mixed 1:1 with PNP if VKA treatment	99^th^ percentile: A. <63% B. <56% C. <49%	Primary APS (106): 83 TAPS and 23 OAPS	A: 67/106 B: 61/106 C: 57/106	Thrombotic control, no APS or inherited thrombophilia (36)	A: 5/36 B: 6/36 C: 3/36	A: 10.7 (3.83-29.7) B: 6.78 (2.60-17.7) C: 12.8 (3.70-44.3)	A: <0.0001 B: 0.0001 C: 0.0001
					SLE patients without thrombotic history (37)	A: 17/37 B: 19/37 C: 17/37	A: 2.02 (0.95-4.30) B: 1.28 (0.61-2.72) C: 1.37 (0.65-2.90)	A: 0.07 B: 0.51 C: 0.42
Ramirez (2021)	Inhibition (%) of ETP (CAT) with Protac and rhAPC using PPP, mixed 1:1 with PNP if VKA treatment	99^th^ percentile of 100 healthy subjects: rhAPC <56%; Protac <63%	Secondary APS (24)	Not specified	SLE with aPL without thrombotic history (26)	Not specified	Not available	Not significant
					SLE without aPL (n = 14)			
					Isolated SLE (n=37)			
**B. Case-control studies**								
Green (2012)	Normalised ratio of AUC from TG assay with and without Protac in PPP	5th percentile of 35 healthy subjects	TAPS without anticoagulation (17)	11/17	Patients with history of thrombosis and FV Leiden (19)	19/19	0.045 (0.002-0.88)	0.04
					Patients with history of thrombosis and with Protein C or S deficiency (9)	8/9	0.23 (0.02-2.3)	0.21
					Thrombotic control, no APS or inherited thrombophilia (42)	10/42	5.87 (1.73-19.9)	0.005

Abbreviations: aPC-r, activated protein C resistance; aPL, antiphospholipid antibodies; APS, antiphospholipid syndrome; AUC, area under the thrombin generation curve; CAT, calibrated automated thrombography; ETP, endogenous thrombin potential; FV, Factor V; OAPS, obstetric APS; PNP, pooled normal plasma; PPP, platelet poor plasma; rhAPC, recombinant human activated protein C; SLE, systemic lupus erythematosus; TAPS, thrombotic APS; TM, thrombomodulin; TG, thrombin generation; VKA, vitamin K antagonist.

**Figure 2:**
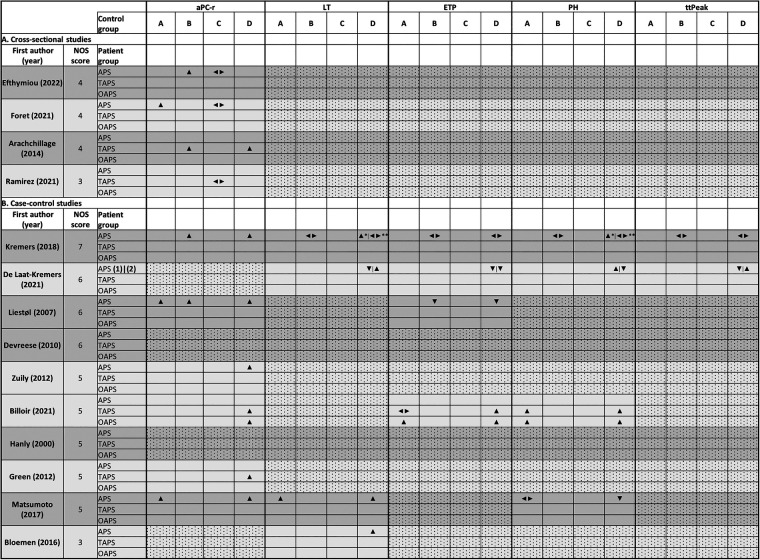
Effect direction plot comparing different outcomes between patient groups and control groups. Explanation: ▴: increased in patient population compared to control population; ▾: decreased in patient population compared to control population; ◄►: no significant difference between patient and control population. Control populations: (**A**): antiphospholipid antibody carriers; (**B**): thrombotic controls and control patients on vitamin K antagonist therapy; (**C**): autoimmune disease control group; (**D**): presumably healthy controls. *Thrombin generation with 1 pM TF, **5 pM TF; (1) developmental cohort, (2) validation cohort. Abbreviations: aPC-r, activated protein C resistance; APS, antiphospholipid syndrome; ETP, endogenous thrombin potential; LT, lag time; NOS, Newcastle-Ottawa Score; OAPS, obstetric APS; PH, peak height; TAPS, thrombotic APS; ttPeak, time to peak.

As a second outcome, other TG parameters were defined such as PH or thrombin peak, ETP or AUC, lag time (LT), and time to peak (ttPeak) which can be derived from the thrombogram (12). These parameters can be reported as absolute results, but can also be normalised by dividing the patient result by a PNP result of the same run, reducing inter-run variability (15). Seven of the 14 retrieved articles described at least one of the parameters LT, ETP, ttPeak, or PH (Table 5) (38–41, 45–47). Only two studies normalised their data against PNP (41, 46). None of the seven studies reported reference values for the separate parameters. Therefore, only continuous data between patient and control groups could be compared. Kremers et al. could not demonstrate a significant difference in LT, ETP, PH, or ttPeak between APS patients and control groups using CAT with 5.0 pM TF as a trigger (45). When using 1.0 pM TF, no significant differences were observed comparing APS patients treated with VKA compared to a VKA-treated control group, although LT and PH were significantly higher in APS patients without VKA treatment compared to healthy controls (45). On the other hand, the same group found conflicting results in a more recent study (40). They used a developmental cohort consisting of 31 APS patients and 66 healthy controls to set up a neural network including TG and thrombin dynamics parameters. In this cohort, it was observed that APS patients had a shorter LT and ttPeak, decreased ETP and increased PH. In a second cohort, they also found a decreased ETP, and PH, but an increased LT, and ttPeak in APS patients compared to controls. In the second cohort, APS patients on VKA treatment were included, while they were excluded in the developmental cohort. Despite these discrepancies between both cohorts, the neural network was able to differentiate between APS patients and non-APS patients with an accuracy ranging from 73% to 93% depending on the control population considered (40). Liestøl et al. demonstrated a lower normalised ETP in VKA-treated LA-positive APS patients compared to VKA-treated controls (46). Interestingly, LA-positive controls without clinical APS criteria also showed a reduced normalised ETP compared to healthy controls (*p* < 0.001) (46). In contrast, Billoir et al. demonstrated a higher ETP for both thrombotic and obstetric APS patients compared to healthy controls using the CAT method and 1.0 pM TF as an activator (38). When compared to aPL-positive controls (without clinical APS criteria), only obstetric APS patients had significantly higher ETP values, whereas thrombotic APS patients did not. They also showed that thrombotic and obstetric APS patients had a higher PH compared to both healthy controls and aPL-carriers (38). Devreese et al. previously demonstrated that a ratio of PH/LT instead of assessing the parameters separately might be more useful (25). In the study included here, they reported a pilot study that demonstrated that LA-positive thrombotic APS patients displayed a lower PH/LT ratio compared to LA-positive controls, thrombotic controls, and healthy controls (41). Furthermore, Matsumoto et al. described a longer LT in ten LA-positive APS patients compared to a LA-positive control group, but with similar PH levels (47). Bloemen et al. also described a longer LT in APS patients compared to healthy controls, although the patient and control group only consisted of five subjects each (39). Results were summarised in an effect direction plot in Figure 2.

**Table 5:** An overview of thrombin generation parameters.

**First author (year)**	**Parameter (unit)**	**Patient group (*n*)**	**Patient results**	**Control group (*n*)**	**Control results**	**P-value**	
Kremers (2018)	LT (min) ETP (nM●min) PH (nM) ttPeak (min)	A: VKA-treated APS (50)	Graphical representation only	1. VKA-treated controls (31)	Graphical representation only	A-1: n.s.	
		B: APS without VKA (30)		2. Healthy controls (45)		B-2: LT longer (p<0.001) and PH higher (p<0.01) in APS when using 1 pM TF, not when using 5 pM TF. ETP and ttPeak n.s.	
De Laat – Kremers (2021)	LT (min) ETP (nM●min) PH (nM) ttPeak (min)	A: Development cohort: APS (31)	Graphical representation only	1. Development cohort: Healthy controls (66)	Graphical representation only	A-1: APS: shorter LT (p<0.01) and ttPeak, decreased ETP and increased PH compared to controls (p<0.05)	
		B: Validation cohort: APS (42)	Not available	2. Validation cohort: Healthy controls (38)	Not available	B-2: APS: increased LT, decreased ETP, longer ttPeak, and decreased PH in APS patients compared to controls (p<0.05)	
Liestøl (2007)	ETP, w/o aPC (normalised)***	A: VKA-treated APS (34)	0.63 (0.59-0.66)	1. VKA-treated controls (38)	0.73 (0.70-0.77)	A-1: <0.001	
	ETP, with aPC (normalised)***		3.69 (2.38-4.50)		0.17 (0.13-0.28)	A-1: Not reported	
	ETP, w/o aPC (normalised)***	B: APS without VKA (18)	0.94 (0.83-1.00)	2. Healthy controls (53)	1.01 (0.96-1.05)	B-2: <0.001	
	ETP, with aPC (normalised)***		2.61 (2.02-5.04)		0.58 (0.54-0.72)	B-2: Not reported	
	ETP, w/o aPC (normalised)***	Not applicable	Not applicable	3. LA-positive (29)	0.85 (0.81-0.93)	Not reported	
	ETP, with aPC (normalised)***				1.92 (1.22-2.81)		
Devreese (2010)	PH/LT ratio (nM/min)*	A: TAPS (8)	29.8 +/- 33.6	1. LA-positive (8)	90.7 +/- 29.7	A-1: 0.003	
				2. Thrombotic controls (21)	128 +/- 48.5	A-2: 0.0002	
				3. Healthy controls (25)	170 +/- 46.4	A-3: <0.0001	
Billoir (2021)	LT (min)*	A: TAPS (19)	Not reported	1. aPL-carriers (11)	13.6 +/- 3.9	A/B-1: Not reported	
	ETP (nM.min)**		1265 (956-1741)		Not reported	A-1: 0.08	B-1: 0.002
	PH (nM)**		153 (109-215)		Not reported	A-1: 0.048	B-1: 0.001
	LT (min)*	B: OAPS (11)	Not reported	2. Healthy controls (25)	4.89 +/- 1.65	A/B-2: Not reported	
	ETP (nM.min)**		1863 (1434-2080)		808 (756-853)	A-2: <0.001	B-2: <0.001
	PH (nM)**		254 (232-289)		78 (74-86)	A-2: <0.001	B-2: <0.001
Matsumoto (2017)	LT (min)*	A: LA-positive APS (10)	28.8 +/- 11.8	1. LA-positive (10)	12.5 +/- 7.7	A-1: <0.01	
	PH (nM)*		158 +/- 99		158 +/- 75	A-1: Not reported	
	LT (min)*	Not applicable	Not applicable	2. Healthy controls (25)	4.5 +/- 0.3	A-2: <0.01	
	PH (nM)*				362 +/- 23	A-2: Not reported	
Bloemen (2016)	LT (s)**	APS (5)	6.0 (5.15-7.85)	Healthy controls (5)	2.0 (1.75-2.25)	0.008	

^*^ Mean +/- standard deviation,

^**^ Median (IQR),

^***^ Median (95% CI);

Abbreviations: aPC, activated protein C; aPL, antiphospholipid antibodies APS, antiphospholipid syndrome; CI, confidence interval; ETP, endogenous thrombin potential; IQR, interquartile range; LA, lupus anticoagulant; LT, lag time; n.s., not significant; OAPS, obstetric APS; PH, peak height; TAPS, thrombotic APS; TF, tissue factor; ttPeak, time to Peak; VKA, vitamin K antagonist; w/o, without

The outcomes of one study could not be included in one of the proposed groups (44). They used a chromogenic substrate to determine the inhibition of TG by comparing the thrombin generated in patients to healthy controls and expressing results as standard error of mean or z-score. Inhibition of the *in vitro* TG was more significant in APS patients (*n* = 29) compared to control patients without thrombotic or obstetric complications of APS (*n* = 30). When applying a cut-off of |z| = 2, the OR for APS diagnosis was 5.43 (95% CI 1.76–16.8) (44).

## Discussion

The diagnosis of APS predominantly relies on a combination of laboratory assays to measure the presence of aPL. However, these laboratory tests still show methodological and diagnostic shortcomings and a lack of standardisation (50). The laboratory tests for the detection of aPL comprise clotting-based assays for the detection of LA and solid-phase immunoassays for the detection of aCL and a*β*2GPI IgG/IgM antibodies (3). TG assays measure the dynamic process of *in vitro* thrombin generated over time and offer a more global assessment of the coagulation compared to traditional coagulation assays (9). In addition, several studies have investigated the role of TG as a new tool to assess coagulation in patients with a wide variety of coagulation disorders (11, 14). This review was performed to assess if TG assays could be used as a diagnostic tool for, on one hand, the laboratory diagnosis of APS patients and, on the other hand, for identifying APS patients at high risk for recurrent clinical manifestations.

Our review identified 1,160 records when searching the key concepts “thrombin generation” and “antiphospholipid syndrome”. Fourteen articles were included that demonstrated TG data in APS patients. All included publications described retrospective case-control or cross-sectional studies. Interestingly, most of the studies (10/14) measured aPC-r in APS patients using the TG method. Resistance to aPC occurs due to a decreased inhibition of activated coagulation factor V (FVa) by aPC and is an important risk factor for venous thrombosis. aPC-r may occur due to various causes, either inherited (e.g., FV Leiden) or acquired (e.g., use of oral contraceptives) (51). Traditionally, laboratory tests to screen for the aPC pathway are based on the activated partial thromboplastin time (aPTT). In this test, aPC-r is considered when the prolongation of the aPTT by adding aPC is less than expected (52). However, interpretation of aPTT-based aPC-r can be complicated by a prolonged aPTT at baseline, often observed in aPL-positive patients (53). Alternatively, TG-based aPC-r testing could be performed in these patients to overcome the issues associated with aPTT-based aPC-r tests. Furthermore, TG assays might identify patients with resistance to aPC-r that cannot be detected with the traditional assays (54). Nevertheless, there is a need for standardisation of TG-based aPC-r assays, since methodological variation between studies is large. Variation in TG-based aPC-r assays occurs mostly on two levels. First, an exogenous substance needs to be added to the TG reaction to evaluate the aPC pathway as the amount of endogenous aPC formed is too low in a standard TG assay (14). In this review, three exogenous substances were described, namely aPC, Protac®, and TM. Both Protac® (enzymatic) and TM (thrombin cofactor) lead to endogenous protein C activation meaning that the function of endogenous protein C is examined in contrast to when exogenous aPC is added. Secondly, different strategies can be used to compare TG before and after adding the substance for aPC pathway evaluation. Generally, a ratio of the PH or ETP, either normalised with PNP or not, before and after adding the aPC substance is assessed.

The diversity in TG-based aPC-r methods and reporting of the results complicated the general interpretation of the studies in this review, although some conclusions may be drawn. It appears that aPC-r is higher in patients with thrombotic APS compared to healthy controls, but also compared to thrombotic controls without inherited thrombophilia. This also seems to be true for obstetric APS patients, although only two studies analysed obstetric APS patients as a separate group and the total number of patients was relatively small (36, 38). Treatment with VKA did not influence the interpretation as also VKA-treated APS patients showed higher aPC-r, both compared to VKA-treated control groups and healthy controls. Most studies did however attempt to correct VKA-related coagulation factor deficiencies by 1 : 1 mixing of patient plasma with PNP. Interestingly, in one study a VKA-treated control group showed significantly higher aPC-r compared to healthy controls when using Protac®, but not when using aPC even after 1:1 mixing of the samples with PNP (35). The same trend was observed in two other studies, where aPC-r was significantly higher in VKA-treated controls compared to healthy controls when using TM (45) but not with aPC (46). This may be explained by the impaired production of endogenous protein C due to VKA, suggesting that it might be useful to assess underlying aPC-r using aPC instead of TM or Protac® in VKA-treated individuals. Tightly matching patients and controls for anticoagulant treatment should be imperative in investigating the value of TG in APS patients. Unfortunately, only limited information on anticoagulant treatment was provided in the included publications which is a limitation in this review. Three studies included anticoagulant-matched controls. Those studies were matched based on anticoagulant type, but not on intensity of treatment (35, 45, 46).

The prevalence and degree of aPC-r does not seem to differ between patients with primary or secondary APS and patients with SLE even without circulating aPL or a history of clinical manifestations of APS (36, 37, 42, 48). SLE patients with aPC-r might be at higher risk for thrombosis compared to those without aPC-r. This was outside the scope of the review and should be addressed by prospective studies. It is known that the prevalence of circulating non-criteria aPL such as antiphosphatidylserine/prothrombin antibodies (aPS/PT) is higher in patients with AID compared to the general population (55). Presence of these aPL might partially explain comparable aPC-r between APS and SLE (or other AID) patients (56). This raises the question whether the observed aPC-r is an *in vitro* finding due to the presence of aPL or really associated with an increased risk of thrombo-embolic complications. Of note, other mechanisms than presence of aPL leading to impaired protein C activation might be present in AID patients (57).

Information on ethnicity of the included cases was not available in all but one publication. Nevertheless, information regarding ethnicity might be important, since various studies have demonstrated ethnic variation in coagulation parameters and risk for venous and/or arterial thrombosis (58, 59).

In only a few studies included in this review, thrombogram-derived parameters were reported, and mostly in a selective manner introducing considerable selection bias. In most studies no normalisation procedure of data was performed which might lead to a decreased standardisation and comparability between studies (15). Compared to healthy controls, only two studies reported a prolonged LT in APS patients (39, 47) and two studies were conflicting as they each included two patient populations with different LT results (40, 45). In addition, PH and ETP values were highly variable and inconsistent results were reported between studies. This discrepancy may partially be explained by the hypothesis that distinct aPL profiles may be associated with different TG profiles (60). Furthermore, to fairly evaluate the predictive ability of TG in relation to the risk of thrombotic or obstetric manifestations, it is necessary to calculate an OR, or to investigate the sensitivity, specificity, and positive and negative predictive values of the assay. Unfortunately, this was impossible because none of the studies reported a predefined cut-off value for any of the TG-derived parameters. Moreover, it might be more interesting to combine different TG parameters for the diagnostic evaluation of APS patients. Devreese et al. showed that the ratio of PH/LT could partially discriminate LA-positive APS patients from LA-positive patients without thrombosis although this has not been verified in an independent study (41). The study of de Laat-Kremers et al. showed that combining TG parameters and thrombin dynamics might accurately identify APS patients using an artificial intelligence approach (40), but this has to be verified in independent larger cohorts. Furthermore, thrombin dynamics is a technique that is not routinely available (40). It cannot be excluded that TG might be useful as a diagnostic marker as aPL showed direct influence on the TG profile based on spiking experiments (25, 56). This review has demonstrated that there is a lack of evidence on how deviant TG results correlate with a higher thrombotic or obstetric risk in APS patients as no prospective studies were available.

The total amount of thrombin activity over time can be measured using multiple methods, including fluorogenic and chromogenic substrate-based TG methods. Although both methods measure TG, there are significant methodological differences between the two assays. These differences originate from the substrates themselves and the sample preparation needed for each substrate. Fluorogenic substrate-based assays utilise the 7-Amino-4-methylcoumarin (AMC) fluorophore, whereas the chromogenic substrate-based assays use the para-nitroaniline (pNA) chromophore (61, 62). Chromogenic substrates require defibrinated plasma since fibrin can cause turbidity which is known to interfere with the absorption (61). However, the removal of fibrinogen has a profound effect on the TG curves by reducing the PH and increasing the formation of thrombin-α_2_M complexes (62). Finally, the onset of TG is significantly faster in TG assays that utilise a chromogenic substrate compared to assays using a fluorogenic substrate (61). These differences make the TG data from chromogenic and fluorogenic substrate-based techniques hard to compare. Even within the fluorogenic substrate-based methods, ST Genesia® and CAT methodologies showed poor agreement for detecting aPC-r, although a different methodology was used for protein C activation (TM and Protac® or recombinant aPC, respectively) (36). Another study investigating patients with liver disease also demonstrated significant differences between ST Genesia® and CAT analysis (63). Further studies are needed to investigate whether automated TG analysers such as ST Genesia® are adequate for replacing the traditional CAT methodology.

Other differences in the TG protocol also influenced the comparability between studies. Although most included studies in this review used largely identical CAT-TG protocols, four studies used lower TF concentrations. At low TF concentrations, coagulation factors from both the intrinsic and extrinsic pathways influence the TG assay (64), whereas at high TF concentrations, the TG assays are only influenced by the factors of the extrinsic pathway (64). Furthermore, the addition of synthetic phospholipids has been shown to strongly influence all TG parameters (65). Therefore, differences in phospholipid concentration could severely impact the comparability of studies. Overall, following a standardised protocol for conducting and reporting TG research is very important to fairly compare outcomes between different studies. The International Society on Thrombosis and Haemostasis – Scientific and Standardization Committee on Lupus Anticoagulant/Antiphospholipid Antibodies recently published recommendations on the measurement of TG, aiming for an increased standardisation (15), in addition to previous guidelines on platelet-dependent TG (66). No formal heterogeneity assessment was performed, but we can informally conclude that the studies included in this review are extremely heterogenous. While the development of automated TG systems should increase the inter-laboratory harmonisation and standardisation of the TG assay, efforts should be undertaken to follow the international recommendations (15, 66). Recommendations on TG-based aPC-r measurement could benefit harmonisation, as the currently investigated methods are very heterogeneous across studies.

In addition, this study only included patients with APS, diagnosed based on the presence of the aPL that are included in the Sydney classification criteria (LA, aCL, and a*β*2GPI) (1). Information on the assays that were used for aPL detection were not extracted from the studies, although APS classification may depend on the type of assay used (67, 68). Whether including non-classification aPL such as aPS/PT may help to uniformly characterise and classify APS patients or might help to decrease the heterogeneous outcomes observed in this systematic review, is questionable. The more that in the upcoming new classification criteria aPS/PT is not included (69). However, for diagnostic reasons, aPS/PT might help in subpopulations, such as those where LA measurement is hampered by interference of anticoagulation. We acknowledge that there is no gold standard for diagnosis of APS and we rely on classification criteria for conducting research in APS patients. Nevertheless, a prospective study not included in this review identified TG-based aPC-r as a higher risk factor for thrombosis in a population of patients with APS and/or SLE and aPL carriers, compared to the traditional Sydney criteria aPL (33). However, standard-of-care anticoagulant or antiplatelet therapy was initiated in patients based on the treating physicians' assessment introducing bias as patients with a higher risk profile, including the presence of (multiple) aPL, will most likely lead to a higher level of anticoagulation treatment in those patients and thus lower the incidence of thrombosis. This shows that there are both advantages and disadvantages to limiting systematic reviews to studies with patients categorised according to the APS classification criteria as the population of interest.

The goal of this review was to investigate the value of TG assays in the diagnosis and risk stratification of APS. No prospective cohort studies were identified in this review and therefore no information could be synthesised on the obstetric or thrombotic risk associated with abnormal TG patterns in primary and secondary APS patients. However, higher aPC-r values and aPC-r prevalence are observed in APS patients compared to healthy and thrombotic controls, but the diagnostic and prognostic value is unclear compared to current diagnostic strategies. Results of other thrombogram-derived parameters such as LT and PH were conflicting across studies and more research is needed to identify their potential role in APS diagnosis. Publications on TG studies in APS were very heterogeneous in the applied TG methodology, preanalytical variables, and result description. Following the available guideline documents for reporting TG studies might improve harmonisation. Additional guidelines are needed for selection of the adequate TG methodology in APS studies.

## Data Availability

The original contributions presented in the study are included in the article/**Supplementary Material**, further inquiries can be directed to the corresponding author.
