# Supplementary material for: Application of the thrombin generation assay in patients with antiphospholipid syndrome: A systematic review of the literature
Source: Front Cardiovasc Med. 2023 Mar 28;10:1075121. doi: 10.3389/fcvm.2023.1075121 (PMC10089302; doi:10.3389/fcvm.2023.1075121)
Supplement: Supplementary file 1 [file Datasheet1.pdf]

Supplemental Figure 1: Description of the full search strategy via MEDLINE (A) and Embase (B)

A. MEDLINE (via PubMed interface)

Concept 1: antiphospholipid syndrome

"Antiphospholipid Syndrome"[MeSH Terms] OR "antibodies, antiphospholipid"[MeSH Terms] OR "Hughes Syndrome"[Title/Abstract] OR "Antiphospholipidsyndrome"[Title/Abstract] OR "Asherson's syndrome"[Title/Abstract] OR "APLS"[Title/Abstract] OR "OAPS"[Title/Abstract] OR "Antiphospholipid"[Title/Abstract] OR "Anti-phospholipid"[Title/Abstract] OR "Lupus anticoagulant"[Title/Abstract] OR "Lupus anticoagulans"[Title/Abstract] OR "antibeta 2 glycoprotein*"[Title/Abstract] OR "anti beta 2 glycoprotein*"[Title/Abstract] OR "anti beta2 glycoprotein*"[Title/Abstract] OR "antibeta2 glycoprotein*"[Title/Abstract] OR "antibeta2glycoprotein*"[Title/Abstract] OR "anti beta2gp*"[Title/Abstract] OR "antibeta2gp*"[Title/Abstract] OR ("beta 2 glycoprotein*"[Title/Abstract] AND "antibod*"[Title/Abstract]) OR ("beta2 glycoprotein*"[Title/Abstract] AND "antibod*"[Title/Abstract]) OR "Anti-cardiolipin"[Title/Abstract] OR "Anticardiolipin"[Title/Abstract] OR ("cardiolipin"[Title/Abstract] AND "antibod*"[Title/Abstract]) OR "anti-fii"[Title/Abstract] OR ("FII"[Title/Abstract] AND "antibod*"[Title/Abstract]) OR "Anti-prothrombin"[Title/Abstract] OR "Antiprothrombin"[Title/Abstract] OR ("prothrombin"[Title/Abstract] AND "antibod*"[Title/Abstract]) OR "anti phosphatidylserine*"[Title/Abstract] OR "antiphosphatidylserine*"[Title/Abstract] OR ("phosphatidylserine*"[Title/Abstract] AND "antibod*"[Title/Abstract]) OR "Anti-domain 1"[Title/Abstract] OR ("domain 1"[Title/Abstract] AND "antibod*"[Title/Abstract]) OR "Anti-domain I"[Title/Abstract] OR ("domain I"[Title/Abstract] AND "antibod*"[Title/Abstract])

Concept 2: thrombin generation

"Thrombography"[Title/Abstract] OR "Thrombin generation"[Title/Abstract] OR "Thrombin formation"[Title/Abstract] OR "CAT"[Title/Abstract] OR "Lag time"[Title/Abstract] OR "Endogenous thrombin potential"[Title/Abstract] OR "Time to peak"[Title/Abstract] OR "Peak height"[Title/Abstract] OR "Peak time"[Title/Abstract] OR "Time to peak"[Title/Abstract] OR "Thrombin dynamics"[Title/Abstract] OR "ETP"[Title/Abstract] OR "Ttpeak"[Title/Abstract] OR "Thrombinoscope"[Title/Abstract] OR "thrombogram"[Title/Abstract]

Combination of concepts: (Concept 1) AND (Concept 2)

No filters were applied.

B. Embase (via embase.com interface)

Concept 1: antiphospholipid syndrome

'antiphospholipid syndrome'/exp OR 'antiphospholipid syndrome' OR 'cardiolipin antibody'/exp OR 'cardiolipin antibody' OR 'phospholipid antibody'/exp OR 'phospholipid antibody' OR 'hughes syndrome':ti,ab,kw OR 'antiphospholipidsyndrome':ti,ab,kw OR 'asherson syndrome':ti,ab,kw OR 'apls':ti,ab,kw OR 'oaps':ti,ab,kw OR 'antiphospholipid':ti,ab,kw OR 'anti-phospholipid':ti,ab,kw OR 'lupus anticoagulant':ti,ab,kw OR 'lupus anticoagulans':ti,ab,kw OR 'antibeta-2 glycoprotein*':ti,ab,kw OR 'anti-beta-2 glycoprotein*':ti,ab,kw OR 'anti-β2-glycoprotein*':ti,ab,kw OR 'anti-β-2-glycoprotein*':ti,ab,kw OR 'antiβ2-glycoprotein*':ti,ab,kw OR 'antiβ2glycoprotein*':ti,ab,kw OR 'anti-β2gp*':ti,ab,kw OR 'antiβ2gp*':ti,ab,kw OR ('beta 2 glycoprotein*':ti,ab,kw AND 'antibod*':ti,ab,kw) OR ('β2-glycoprotein*':ti,ab,kw AND 'antibod*':ti,ab,kw) OR ('β-2-glycoprotein*':ti,ab,kw AND 'antibod*':ti,ab,kw) OR 'anti-cardiolipin':ti,ab,kw OR 'anticardiolipin':ti,ab,kw OR ('cardiolipin':ti,ab,kw AND 'antibod*':ti,ab,kw) OR 'anti-fii':ti,ab,kw OR 'anti-prothrombin':ti,ab,kw OR 'antiprothrombin':ti,ab,kw OR ('prothrombin':ti,ab,kw AND 'antibod*':ti,ab,kw) OR ('fii':ti,ab,kw AND 'antibod*':ti,ab,kw) OR 'anti-phosphatidylserine*':ti,ab,kw OR 'antiphosphatidylserine*':ti,ab,kw OR ('phosphatidylserine*':ti,ab,kw AND 'antibod*':ti,ab,kw) OR 'anti-domain 1':ti,ab,kw OR ('domain 1':ti,ab,kw AND 'antibod*':ti,ab,kw) OR 'anti-domain i':ti,ab,kw OR ('domain i':ti,ab,kw AND 'antibod*':ti,ab,kw)

Concept 2: thrombin generation

'Thrombography':ti,ab,kw OR 'Thrombin generation':ti,ab,kw OR 'Thrombin formation':ti,ab,kw OR 'CAT':ti,ab,kw OR 'Lag time':ti,ab,kw OR 'Endogenous thrombin potential':ti,ab,kw OR 'Time to peak':ti,ab,kw OR 'Peak height':ti,ab,kw OR 'Peak time':ti,ab,kw OR 'Time to peak':ti,ab,kw OR 'Thrombin dynamics':ti,ab,kw OR 'ETP':ti,ab,kw OR 'Ttpeak':ti,ab,kw OR 'Thrombinoscope':ti,ab,kw OR 'thrombogram':ti,ab,kw

Combination of concepts: (Concept 1) AND (Concept 2)

No filters were applied.

Supplemental Table 1: Validation of the search strategy with prespecified validation set of references and proof of retrieval.

Specified reference	Validation of idenitification based on search strategy
Regnault et al, Thromb Haemost 2003; 89: 208-2012	**Thrombinography shows acquired resistance to activated protein C in patients with lupus anticoagulants**<br>V. Regnault, S. Béguin, D. Wahl, E. de Maistre, H. Coenraad Hemker and T. Lecompte<br>Thrombosis and haemostasis 2003 Vol. 89 Issue 2 Pages 208-12
Zuily et al. Lupus 2012, 21: 758-760	**Thrombin generation in antiphospholipid syndrome**<br>S. Zuily, K. A. Aissa, A. Membre, V. Regnault, T. Lecompte and D. Wahl<br>Lupus 2012 Vol. 21 Issue 7 Pages 758-760
Liestol et al, J Thromb Haemost 2007; 5: 2204-10	**Activated protein C resistance determined with a thrombin generation-based test is associated with thrombotic events in patients with lupus anticoagulants**<br>S. Liestøl, P. M. Sandset, M. C. Mowinckel and F. Wisløff<br>Journal of thrombosis and haemostasis : JTH 2007 Vol. 5 Issue 11 Pages 2204-10
Devreese et al, Thromb Haemost 2009; 101, 185-196	**Laboratory detection of the antiphospholipid syndrome via calibrated automated thrombography**<br>K. Devreese, K. Peerlinck, J. Arnout and M. F. Hoylaerts<br>Thrombosis and haemostasis 2009 Vol. 101 Issue 1 Pages 185-96
Devreese et al, Blood 2010; 115: 870-78	**Thrombotic risk assessment in the antiphospholipid syndrome requires more than the quantification of lupus anticoagulants**<br>K. Devreese, K. Peerlinck and M. F. Hoylaerts<br>Blood 2010 Vol. 115 Issue 4 Pages 870-8
de Laat-Kremers et al, Throm Res 2021, 203: 142-151	**Deciphered coagulation profile to diagnose the antiphospholipid syndrome using artificial intelligence**<br>R. M. W. de Laat-Kremers, D. Wahl, S. Zuily, M. Ninivaggi, W. Chayouâ, V. Regnault, et al.<br>Thrombosis research 2021 Vol. 203 Pages 142-151

Supplemental Table 2: Data items extracted from identified articles.

Items extracted	Examples/explanation
**General information**:	
- Authors	
- Year of publication	
- Title	
- Country	
- Academic setting	e.g. Tertiary hospital
**Study methodology**:	
- Study design	e.g. case control
- Monocentric/multicentric approach	
- Funding source	
**Study population:**	
- Type APS	e.g. thrombotic APS
- Number of patients	
- Age	
- Sex	
- Ethnicity	
- Comorbidity	e.g. SLE
- Classification criteria applied	e.g. Sapporo criteria
- Thrombosis specification	e.g. venous thrombosis
- Pregnancy morbidity specification	
- Method of recruitment	
- Anticoagulant medication	
**Control population:**	
- Clinical characteristics	
- Number of patients	
- Age	
- Sex	
- Ethnicity	
- Morbidity and comorbidities	
- Method of recruitment	
- Anticoagulant medication	
**Control outcomes:**	
- Type of aPL determined	
- Reported aPL results	
- Applied cut-off value	
- Method of cut-off determination	
**Thrombin generation assay characteristics:**	
- Thrombin generation method applied	e.g. calibrated automated thrombinography
- Thrombin generation instrument used	
- Amount of replicates	e.g. determined in duplicate
- Tissue factor concentration	
- Tissue factor origin	
- Phospholipid concentration	
- Phospholipid origin	
- Non-tissue factor activator (if present)	
- Sample type and preparation	e.g. platelet poor plasma (+ preanalytics)
- Alpha2-macroglobulin correction	
- Description of other reagents	e.g. thrombomodulin
- Report of results	e.g. normalized or absolute
**Outcomes:**	
- Results descriptors	
• Lag time	
• Time to peak	
• ETP/AUC	
• Peak (height)	
• Start tail	
• Velocity index	
• ETP inhibition	
• Other derived parameters	
- Statistical methodology	
- Effect measures (for outcomes APS, thrombosis in APS or obstetric morbidity in APS):	
• Odds ratio (calculated when possible)	
• Likelihood ratio	
• Sensitivity	
• Specificity	
• AUC (ROC)	
• Relative risk	
• Prevalence	

Abbreviations: aPL, antiphospholipid antibodies; APS, antiphospholipid syndrome; AUC, area under the curve; ETP, endogenous thrombin potential; ROC, receiver operating characteristics; SLE, systemic lupus erythematosus

Supplemental Figure 2: Newcastle – Ottawa Scale (NOS) for case control, cohort, and cross-sectional studies.

NEWCASTLE - OTTAWA QUALITY ASSESSMENT SCALE
CASE CONTROL STUDIES

Note: A study can be awarded a maximum of one star for each numbered item within the Selection and Exposure categories. A maximum of two stars can be given for Comparability.

Selection

1) Is the case definition adequate?
   a) yes, with independent validation ✵
   b) yes, eg record linkage or based on self reports
   c) no description

2) Representativeness of the cases
   a) consecutive or obviously representative series of cases ✵
   b) potential for selection biases or not stated

3) Selection of Controls
   a) community controls ✵
   b) hospital controls
   c) no description

4) Definition of Controls
   a) no history of disease (endpoint) ✵
   b) no description of source

Comparability

1) Comparability of cases and controls on the basis of the design or analysis
   a) study controls for _______________ (Select the most important factor.) ✵
   b) study controls for any additional factor ✵ (This criteria could be modified to indicate specific control for a second important factor.)

Exposure

1) Ascertainment of exposure
   a) secure record (eg surgical records) ✵
   b) structured interview where blind to case/control status ✵
   c) interview not blinded to case/control status
   d) written self report or medical record only
   e) no description

2) Same method of ascertainment for cases and controls
   a) yes ✵
   b) no

3) Non-Response rate
   a) same rate for both groups ✵
   b) non respondents described
   c) rate different and no designation

NEWCASTLE - OTTAWA QUALITY ASSESSMENT SCALE
COHORT STUDIES

Note: A study can be awarded a maximum of one star for each numbered item within the Selection and Outcome categories. A maximum of two stars can be given for Comparability

Selection

1) Representativeness of the exposed cohort
   a) truly representative of the average _______________ (describe) in the community ✵
   b) somewhat representative of the average ______________ in the community ✵
   c) selected group of users eg nurses, volunteers
   d) no description of the derivation of the cohort

2) Selection of the non exposed cohort
   a) drawn from the same community as the exposed cohort ✵
   b) drawn from a different source
   c) no description of the derivation of the non exposed cohort

3) Ascertainment of exposure
   a) secure record (eg surgical records) ✵
   b) structured interview ✵
   c) written self report
   d) no description

4) Demonstration that outcome of interest was not present at start of study
   a) yes ✵
   b) no

Comparability

1) Comparability of cohorts on the basis of the design or analysis
   a) study controls for _____________ (select the most important factor) ✵
   b) study controls for any additional factor ✵ (This criteria could be modified to indicate specific control for a second important factor.)

Outcome

1) Assessment of outcome
   a) independent blind assessment ✵
   b) record linkage ✵
   c) self report
   d) no description

2) Was follow-up long enough for outcomes to occur
   a) yes (select an adequate follow up period for outcome of interest) ✵
   b) no

3) Adequacy of follow up of cohorts
   a) complete follow up - all subjects accounted for ✵
   b) subjects lost to follow up unlikely to introduce bias - small number lost - > ____ % (select an adequate %) follow up, or description provided of those lost) ✵
   c) follow up rate < ____% (select an adequate %) and no description of those lost
   d) no statement

NEWCASTLE - OTTAWA QUALITY ASSESSMENT SCALE
(adapted for cross-sectional studies)

Selection: (Maximum 3 stars)

1) Representativeness of the sample:
   a) Truly representative of the average in the target population. ✵ (all subjects or random sampling)
   b) Somewhat representative of the average in the target population. ✵ (non-random sampling)
   c) Selected group of users.
   d) No description of the sampling strategy.

2) Non-respondents:
   a) Comparability between respondents and non-respondents characteristics is established, and the response rate is satisfactory. ✵
   b) The response rate is unsatisfactory, or the comparability between respondents and non-respondents is unsatisfactory.
   c) No description of the response rate or the characteristics of the responders and the non-responders.

3) Ascertainment of the exposure (risk factor):
   a) Validated measurement tool. ✵
   b) Non-validated measurement tool, but the tool is available or described.
   c) No description of the measurement tool.

Comparability: (Maximum 2 stars)

1) The subjects in different outcome groups are comparable, based on the study design or analysis. Confounding factors are controlled.
   a) The study controls for the most important factor (select one). ✵
   b) The study control for any additional factor. ✵

Outcome: (Maximum 2 stars)

1) Assessment of the outcome:
   a) Independent blind assessment. ✵
   b) Record linkage. ✵
   c) Self report.
   d) No description.

2) Statistical test:
   a) The statistical test used to analyze the data is clearly described and appropriate, and the measurement of the association is presented, including confidence intervals and the probability level (p value). ✵
   b) The statistical test is not appropriate, not described or incomplete.

Supplemental Table 3: Reasons for exclusion of articles that had undergone full-text evaluation

N°	**Author** (first)	**year**	**Title**	**Journal**	**Vol.**	**Issue**	**Pages**	**Reason for exclusion**
1	Boeer et al. (1)	2013	Thrombin generation as marker to estimate thrombosis risk in patients with abnormal test results in lupus anticoagulant routine diagnostics	Thrombosis Journal	11	24	/	Not the study population of interest: cross-sectional study of 64 patients tested for LA, not clear whether patients that were included had APS. CAT was measured with two different PL concentrations and TG parameters were evaluated regarding history of thrombosis.
2	Brandt et al. (2)	1993	Antibodies to Beta2-glycoprotein I inhibit phospholipid dependent coagulation reactions	Thrombosis and Haemostasis	70	4	598-602	Not the study design of interest: Evaluation of various monoclonal antibodies in a TG assay.
3	Devreese et al. (3)	2009	Laboratory detection of the antiphospholipid syndrome via calibrated automated thrombography	Thrombosis and Haemostasis	101	1	185-196	Not the study population of interest: Case-control study including LA-positive and LA-negative patients, not clear whether patients that were included had APS. Normalized PH/LT ratio was compared to LA presence. Also evaluation of monoclonal aβ2GPI in CAT.
4	Lean et al. (4)	2006	The effects of tissue factor pathway inhibitor and anti-beta-2-glycoprotein-I IgG on thrombin generation	Haematologica	91	10	1360-1366	Not the study population/design of interest: Case-control study comparing influence of IgG fractions isolated from patients with and without presence of aPL on a TG assay, not clear whether patients that were included had APS.
5	Lecompte et al. (5)	2007	Hypercoagulability resulting from opposite effects of lupus anticoagulants is associated strongly with thrombotic risk	Haematologica	92	5	714-715	Not the study design of interest (article type): letter to the editor. Case-control study investigating TG based aPCsr and IC50-aPC in APS and non-APS patients.
6	Liestøl et al. (6)	2007	Decreased anticoagulant response to tissue factor pathway inhibitor type 1 in plasmas from patients with lupus anticoagulants	British Journal of Haematology	136	1	131-137	Not the study design of interest: Case-control study including patients and controls with and without APS/LA presence, investigating influence of purified IgG fractions on CAT parameters in PNP and effect of LA on TFPI function.
7	Radin et al. (7)	2021	Cerebrovascular events in patients with isolated anti-phosphatidyl-serine/prothrombin antibodies	Immunologic Research	69	4	372-377	Not the study population of interest: cross-sectional study of 42 patients persistent positive for aPS/PT and negative for aβ2GPI/aCL. TG parameters were compared between LA positive and negative patients, not based on APS diagnosis.
8	Regnault et al. (8)	2003	Thrombinography shows acquired resistance to activated protein C in patients with lupus anticoagulants	Thrombosis and Haemostasis	89	2	208-212	Not the study population of interest: series of 8 consecutive LA-positive patients were tested for TG, investigating effect of LA on TG parameters. No comparison between APS patients and patients without APS was performed.
9	Sheng et al. (9)	2001	Detection of 'antiphospholipid' antibodies: a single chromogenic assay of thrombin generation sensitively detects lupus anticoagulants, anticardiolipin antibodies, plus antibodies binding Beta2-glycoprotein I and prothrombin	Clilnical & Experimental Immunology	124	3	502-508	Not the study design of interest: Case-control study including 13 APS patients and 33 non-APS patients. Investigates influence of patient-derived and monoclonal antibodies on TG. Further examines influence of patient plasma on TG in PNP by mixing both and assessing % change in OD compared to baseline TG measured in PNP. No direct interpretation of TG in patient plasma and therefore not included in data extraction.
10	Slavik et al. (10)	2016	Possibility of coagulation system activation determination with tissue factor in pregnancy complications	Clinical Laboratory	62	10	1851-1856	Not the study population of interest: Case-control study in which patients with normal pregnancy and abnormal pregnancy are compared for TG among others. Relationship with APS cannot be derived from the article.
11	Zuily et al. (11)	2020	Anti–domain I beta-2-glycoprotein I antibodies and activated protein C resistance predict thrombosis in antiphospholipid syndrome: TAC(I)T study	Journal of applied laboratory medicine	5	6	1242-1252	Not the study population of interest: prospective cohort study including 137 consecutive patients with aPL or SLE. Study compared hazard ratios for occurrence of thrombosis between presence of aPC resistance, aDI, and (non-)criteria aPL. Hazard ratios were calculated based on the data from all patients, but no separate analysis was done to determine hazard ratios for the APS patients population. Therefore data specific for APS patients could not be extracted.
12	Zuily et al. (12)	2013	Superficial vein thrombosis, thrombin generation and activated protein C resistance as predictors of thromboembolic events in lupus and antiphospholipid patients. A prospective cohort study	Thrombosis Research	132	1	e1-e7	Not the study population of interest: prospective cohort study including 92 consecutive patients with aPLs or SLE. Study compared hazard ratios for occurrence of thrombosis between presence of aPC resistance, and criteria aPL. Hazard ratios were calculated based on the data from all patients, but no separate analysis was done to determine hazard ratios for the APS patients population. Therefore data specific for APS patients could not be extracted.

Abbreviations: aβ2GPI, anti-β2-glycoprotein I antibodies; aCL, anticardiolipin antibodies; aDI, anti-domain-1-β2GPI; aPC, activated protein C; aPCsr, aPC sensitivity ratio; aPL, antiphospholipid antibodies; APS, antiphospholipid syndrome; aPS/PT, anti-phosphatidylserine/prothrombin antibodies; CAT, calibrated automated thrombinography; LA, lupus anticoagulant; LT, lag time; OD, optical density; PH, peak height; PL, phospholipid; PNP, pooled normal plasma; SLE, systemic lupus erythematosus; TFPI, tissue factor pathway inhibitor; TG, thrombin generation.

Supplemental Table 4: Characteristics of the included studies

**Study** (first author and year)	**Study design**	**Patient population**	**Patient characteristics:** **Age** (mean y ± SD) **Sex** (% Male)	**Control population**	**Control characteristics:** **Age** (mean ± SD) **Sex** (% Male)	**CAT method**	**Outcome** (normalised/ absolute)	**Result**	**Conclusion**
Arachchillage 2014 (13)	Cross-sectional	Thrombotic APS patients on long term warfarin anticoagulation for VTE (n=51)<br>○ 2/51: Secondary with SLE	Age = 49.4 ± 14.8<br>Sex = 51%	Patients on long term warfarin anticoagulation for VTE (without APS) (n=51)<br><br>Healthy controls (n=51)	Age = 50.0 ± 15.1<br>Sex = 47%<br><br>Age = 41.0 ± 12.1<br>Sex = 47%	PPP (mixed 1:1 with PNP)<br>5.0 pM TF<br>(± APC and Protac)<br>4 µM phospholipids (PPP reagents Stago)	APC resistance (normalised)	Inhibition of ETP by APC or Protac (median (95% CI)):<br>○ Warfarin-treated APS patient: Protac = 66.0% (59.5-72.6); APC = 81.3% (75.2-88.3)<br>○ Warfarin-treated controls: Protac = 80.7% (74.2-87.2); APC = 97.7% (93.6-101.8)<br>○ Healthy controls: Protac = 102% (96.2-108.1); APC = 98.3% (92.2-104.3)	APS patients with vascular thrombosis on long-term warfarin show greater resistance to both exogenous APC and to activation of endogenous protein C by Protac in the CAT-TG assay, than non-APS patients with VTE and normal controls. Non-APS patients also showed greater resistance to activation of endogenous protein C than normal controls, but resistance was greater in the APS patients.
Billoir 2021 (14)	Case control	Thrombotic APS patients (n=19)<br>○ 16/19: Primary APS<br>○ 3/19: Secondary with SLE<br><br>Obstetric primary APS patients (n=11)	Age = 49.5 ± 18.3<br>Sex = 16%<br><br>Age = 36.3 ± 8.8<br>Sex = 0%	Biological APS patients (aPL carriers without APS) (n=11)<br><br>Healthy controls (n=25)	Age = 57.1 ± 19.7<br>Sex = 9%<br><br>Age= N/S<br>Sex = N/S	PPP<br>1.0 pM TF<br>(± APC 6.7 nM)<br>4 µM phospholipids (PPP low reagents Stago)	Lag time, ETP, Peak height (absolute)<br><br>APC resistance (absolute)	Lag time, ETP, peak height (mean ± SD or median(IQR)):<br>○ Thrombotic APS patients: Lag time N/S, ETP= 1265 nM.min (956-1741), peak height= 153 nM (109-215)<br>○ Obstetric APS patients: Lag time N/S, ETP= 1863 nM.min (1434-2080), peak height= 254 nM (232-289)<br>○ Biological APS patients: Lag time=13.6 ± 3.9 min, ETP= N/S, peak height= N/S<br>○ Healthy controls: Lag time= 4.89 ± 1.65 min, ETP= 808 nM.min (756-853), peak height= 78 nM (74-86)<br><br>Activated protein C resistance ratio (aPCr; mean ± SD):<br>○ Thrombotic APS patients: 52.7 ± 16.4%<br>○ Obstetric APS patients: 64.1 ± 14.6%<br>○ Biological APS patients: N/S<br>○ Healthy controls: 27.2 ± 13.8%	CAT-TG showed an increase of global TG (ETP, peak height) in thrombotic and obstetric APS patients compared to controls and biological APS patients. Although, ETP increase was not significant for thrombotic APS compared to aPL carriers (p=0.08). Moreover, TG seems to be higher in obstetric APS patients compared to thrombotic APS patients. In addition, thrombotic and obstetric APS patients had a significant resistance to APC compared to controls.
Bloemen 2016 (15)	Case control	APS patients (n=5)<br>○ VKA-treated (n=3)<br>○ Untreated (n=2)	Age= N/S<br>Sex = N/S	Healthy controls (n=5)	Age= N/S<br>Sex = N/S	PPP<br>5.0 pM TF<br>Inverted erythrocyte membranes as phospholipid source	Lag time (absolute)	Lag time (Median (IQR)):<br>○ APS patients: 6.0 s (5.15–7.85)<br>- VKA-treated: N/S<br>- Untreated: N/S<br>○ Healthy controls: 2.0 s (1.75–2.25)	A consistent prolongation of the lag time was evident for the APS patients compared to normal donors. No difference in lag time was found between VKA-treated and untreated patients.
De Laat-Kremers 2021 (16)	Case control	<u>Developmental cohort:</u><br>APS patients (n=31)<br>○ Thrombotic APS (20/31)<br>○ Obstetric APS (11/31)<br>○ Primary APS (22/31)<br>○ Secondary APS (9/31)<br><br><u>Validation cohort</u>:<br>APS patients (n=42)<br>○ Thrombotic APS (36/42)<br>○ Obstetric APS (6/42)<br>○ Primary APS (40/42)<br>○ Secondary APS (2/42)	Age = 46.0 ± 13.0<br>Sex = 10%<br><br>Age = 47.0 ± 14.0<br>Sex = 33%	<u>Developmental cohort</u>:<br>○ Healthy controls (n=66)<br><br><u>Validation cohort:</u><br>○ Healthy controls (n=38)<br><br>○ Hospital controls (n=93)<br><br>○ AID controls (n=49)<br><br>○ Thrombotic controls (n=39)	Age = 42.0 ± 12.0<br>Sex = 31%<br><br>Age = 54.0 ± 9.0<br>Sex = 39%<br><br>Age = 37.7 ± 7.0<br>Sex = 5%<br><br>Age = 46.0 ± 13.0<br>Sex = 14%<br>Age = 47.0 ± 13.0<br>Sex = 44%	PPP<br>1.0 pM and 5.0 pM TF<br>(± TM)<br>4 µM phospholipids (PPP low/ PPP reagents Stago)	Neural network performance<br><br>Secondary: lag time, time to peak, ETP, peak height (absolute)	<u>Developmental cohort</u> (mean ± SD):<br>○ APS vs healthy controls: Accuracy = 99.8±0.4%, PPV = 100±0%, NPV = 99.7±0.6%, Sensitivity =99.4±1.4%, Specificity = 100.0±0.0%<br><br><u>Validation cohort:</u><br>○ APS vs healthy controls: Accuracy = 90.5±0.9%, PPV = 90.8±1.9%, NPV = 90.2±1.1%, Sensitivity = 91.2±1.2%, Specificity = 89.7±2.3%<br>○ APS vs hospital controls: Accuracy = 79.9±2.7%, PPV = 62.0±N/S%, NPV = 95.0±0.5%, Sensitivity = 91.2±1.2%, Specificity = 74.8±4.2%<br>○ APS vs AID controls: Accuracy = 86.8±2.8%, PPV = 83.1±4.7%, NPV = 91.1±3.1%, Sensitivity = 90.2±4.3%, Specificity = 83.9±5.8%<br>○ APS vs thrombotic controls: Accuracy = 72.8±3.1%, PPV = 67.7±N/S%, NPV = 85.5±1.9%, Sensitivity = 91.7±1.7%, Specificity = 52.6±7.8%	The neural network based on functional coagulation assays (TG and thrombin dynamics) shows high potential for diagnosing APS patients.

Devreese 2010 (17)	Case control	Pilot study:<br>Thrombotic APS patients (n=8)<br><br>Main study:<br>APS patients (n=38)<br>○ Thrombotic APS patients (n=37), of which 3 also obstetric complications<br>○ Obstetric APS patients (n=1)	Age= N/S<br>Sex = N/S<br><br><br>Age= N/S<br>Sex = N/S	Pilot study:<br>○ LA positive controls (without thrombotic manifestations of APS) (n=8)<br>○ Thrombotic controls (n=21)<br>○ Healthy controls (n=25)<br><br>Main study:<br>○ LA positive controls (without clinical manifestations of APS) (n=21)<br>○ Healthy controls (n=50)	Age= N/S<br>Sex = N/S<br><br><br>Age= N/S<br>Sex = N/S<br><br>Age= N/S<br>Sex = N/S<br><br><br>Age= N/S<br>Sex = N/S<br><br><br>Age= N/S<br>Sex = N/S	PPP (mixed 1:1 with PNP)<br>5.0 pM TF<br>1 µM phospholipids	PH/LT ratio (normalised)<br><br>Titer determination for LA.	Pilot study (peak height/Lag time ratio; Median ± IQR):<br>○ Thrombotic APS patients: 29.8±33.6 nM/Min<br>○ LA positive controls: 90.7±29.7 nM/Min<br>○ Thrombotic controls: 128.1±48.5 nM/Min<br>○ Control Group: 169.6±46.4 nM/Min<br><br>Main study:<br>○ LA titers (calculated using peak height/lag time ratio): OR=3.54 (95% CI 1.07-11.67), PPV 79%, NPV 48%, sensitivity 64%, specificity 67%<br>○ aβ2GPI titers (calculated using peak height/lag time ratio): OR=10.0(95% CI 1.07-11.67), PPV 91%, NPV 50%, sensitivity 57%, specificity 88%	LA can be detected using CAT-TG in LA positive APS patients and LA positive controls by calculating the peak height/Lag time ratio. However, the use of CAT-TG as a quantitative LA assay is only partially informative in the prediction of APS patients at risk for thrombosis.
Efthymiou 2022 (18)	Cross-sectional	Primary APS patients (n=106)<br>○ Thrombotic APS patients (n=83)<br>○ Obstetric APS patients (n=23)	Age = 47.2± 14.7<br>Sex = 24%<br>Age = 51.3± 18.4<br>Sex = 30%<br>Age = 44.1± 6.8<br>Sex = 0%	Thrombotic control patients (n=36)<br><br>All Systemic lupus erythematosus (SLE) patients (n=53)<br>○ SLE patients (with aPL and thrombosis → secondary APS) (n=16)<br>○ SLE control patients (with aPL without thrombosis) (n=20)<br>○ SLE control patients (without aPL and thrombosis)(n=17)<br><br>Healthy controls (n=75)	Age = 49.1 ± 15.1<br>Sex = 39%<br><br>Age = 45.6 ± 12.2<br>Sex = 13%<br><br>Age = 48.1 ± 15.9<br>Sex = 25%<br><br>Age = 46.9 ± 11.8<br>Sex = 25%<br><br>Age = 44.5 ± 8.5<br>Sex = 17%	CAT-TG<br>PPP(mixed 1:1 with PNP in anticoagulated patients)<br>5.0 pM TF<br>(± APC 5.0 nM and Protac 0.1 U/ml)<br>4 µM phospholipids (PPP reagents Stago)<br><br>ST-Genesia<br>PPP (mixed 1:1 with PNP in anticoagulated patients)<br>Medium pM TF<br>(± TM (Thromboscreen)	APC resistance (absolute)	ST-Genesia APCr positive (%):<br>○ All APS: 53.8%<br>○ Thrombotic APS: 50.6%<br>○ Obstetric APS: 65.2%<br>○ Triple aPL positive: 66.6%<br>○ Double aPL positive: 44.4%<br>○ Single aPL positive: 50.0%<br>○ ALL SLE: 50.0%<br>○ Thrombotic controls: 8.3%<br><br>CAT-TG APCr positive (%) (APC/Protac):<br>○ All APS: 57.5%/63.2%<br>○ Thrombotic APS: 57.8%/59.0%<br>○ Obstetric APS: 56.5%/78.3%<br>○ Triple aPL positive: 80.0%/93.3%<br>○ Double aPL positive: 58.3%/58.3%<br>○ Single aPL positive: 56.2%/68.8%<br>○ ALL SLE: 59.3%/70.4%<br>○ Thrombotic controls: 16.7%/13.9%	Activated protein C resistance was higher in APS and SLE patients compared to thrombotic controls in all three TG methods. In addition, APCr was greater in triple aPL APS patients compared to double/single aPL patients. Nevertheless, despite the ST-Genesia and the CAT-TG analyser having a broadly similar methodology, the agreement between the different TG methods was only poor to fair and with no clear indication of which analyser or method gives a true reflection of hypercoagulability in these patients.
Foret 2021 (19)	Cross-sectional	Primary and secondary APS patients (n=82)	Age = N/S<br>Sex = N/S	AID controls (>91% SLE) (n=15)<br><br>aPL carriers without clinical manifestations of APS (n=20)	Age = N/S<br>Sex = N/S<br><br>Age = N/S<br>Sex = N/S	PRP<br>Low TF concentration<br>(± APC 25 nM f.c.)	APC resistance (absolute)	APC sensitivity ratio (APCsr; mean ± SD):<br>○ APS patients: 0.583 ± 0.278<br>○ AID controls: Value N/S in article<br>○ aPL carriers: 0.423 ± 0.239<br><br>○ Triple positive aPL test: 0.693 ± 0.219<br>○ Single positive aPL test: 0.437 ± 0.270<br><br>○ LA positive: 0.603 ± 0.251<br>○ LA negative: 0.392 ± 0.210<br>○ aDI positive: 0.648 ± 0.227<br>○ aDI negative: 0.483 ± 0.329<br><br>○ aCL IgG: 0.573 ± 0.273<br>○ aCL IgM: 0.395 ± 0.215<br>○ aβ2GPI IgG: 0.698 ± 0.257<br>○ aβ2GPI IgM: 0.390 ± 0.152	APS patients have a more marked APC resistance compared to aPL carriers. In contrast, no difference was observed in APC sensitivity between APS patients and AID controls. In addition, triple positive, LA positive and aDI positive patients have a higher APC resistance compared to single positive, LA negative and aDI negative patients. Moreover, APC resistance was higher according to the immunoglobulin isotype of aPL.
Green 2012 (20)	Case control	Thrombotic APS (not on anticoagulant therapy > 8 weeks) (n=17)	Age = N/S<br>Sex = N/S	Healthy controls (n=35)<br><br>Thrombotic controls (n=60)<br>○ No inherited thrombophilia (n=42)<br>○ FV Leiden (n=19)<br>○ Protein C/S deficiency (n=9)	Age = 37.0 ± N/S<br>Sex = 54%<br><br>Age = N/S<br>Sex = N/S	PPP<br>1.5 pM TF<br>(± Protac 0.135 U/ml)<br>10 µg/ml phospholipids<br>Polymerization inhibitor	APC resistance (normalised)	Normalised ratio of the area under the curve ratio (AUCnr; Median (IQR)):<br>○ APS patients: 1.1 (0.8-1.4)<br>○ Healthy controls: 2.8 (2.4-4.7)<br><br>aPC-r in 11/17 APS patients and 10/42 thrombotic controls without inherited thrombophilia.	The AUCnr of APS patient was significantly lower compared to healthy controls. In addition, APC resistance was confirmed in 64% of APS patients.
Hanly 2000 (21)	Case control	Primary thrombotic and obstetric APS patients (n=24)	Age = N/S<br>Sex = N/S	Secondary APS patients (with SLE) (n=13)<br><br>SLE patients (with aPL without APS) (n=18)	Age = N/S<br>Sex = N/S<br><br><br>Age = N/S<br>Sex = N/S	PPP<br>Thromboplastin	Thrombin generation inhibition	Thrombin generation inhibition (mean ± SEM Z score):<br>○ Primary APS patients: -3.86 ± 0.48<br>○ Secondary APS patients: -2.47 ± 0.78<br>- All APS patients (n=29): -3.38 ± 0.51<br>○ SLE patients:(-1.23 ± 0.57	Primary APS patients revealed significantly greater inhibition of TG compared to SLE patients. In addition, Patients with core manifestations of APS had significantly greater inhibition compared to aPL carriers without clinical manifestations.

				aPL carriers (without APS or SLE) (n=4)<br><br>Healthy controls (n=12)	Age = N/S<br>Sex = N/S<br><br>Age = N/S<br>Sex = N/S			o aPL carriers: (N/S)<br>- All non APS patients (n=30): -1.42 ± 0.56<br>o Healthy controls: (N/S)	Inhibition of TG is associated with clinical manifestations of APS (OR: 5.43). The strength of this association is higher compared to the traditional laboratory markers for APS, aCL and aβ2GPI IgG antibodies (OR:4.17 and 3.28).
Kremers 2018 (22)	Case control	APS patients (n=80)<br>o VKA-treated APS patients (50/80)<br>o APS patients not on VKA treatment (30/80)<br>o Primary APS patients (54/80)<br>o Secondary APS patients (26/80)<br>o Thrombotic manifestations (n=62)<br>o Obstetric manifestations (n=11)<br>o Obstretic and thrombotic manifestations (n=7)	Age = 45.0± 15.0<br>Sex = 36%<br>Age = 46.0± 13.0<br>Sex = 10%	VKA-treated control subjects (n=31)<br><br>Healthy controls (n=45)	Age and sex matched<br><br>Age and sex matched	PPP<br>1.0/5.0 pM TF<br>(± TM 20 nM f.c.)<br>4 µM phospholipids	APC resistance (absolute)<br><br>lag time, time to peak, ETP, peak height (absolute)	The lag time and peak height (only at 1 pM TF) was significantly increased in APS patients without VKA therapy compared to controls. In addition, the ETP and peak height (only at 5 pM TF) were significantly higher in APS patients with prior thrombosis, compared to APS patients without a history of thrombosis.<br><br>Percent inhibition of peak height by TM (median):<br>o APS patients: 10%<br>- With history of thrombosis: 6%<br>- Without history of thrombosis: 11%<br>o VKA-treated APS patients: 15%<br>o VKA-treated Controls: 35%<br>o Healthy controls: 50%	CAT-TG showed a marked prolongation of the lag time and elevation of the peak height in APS patients. In addition, ETP and peak height were increased in APS patients with prior thrombosis, indicating that elevation of TG might be a prothrombotic mechanism in APS.<br><br>APS patients had a significant resistance to TM compared to controls. Moreover, TM resistance was significantly more pronounced in patients with prior thrombosis compared to APS patients without prior thrombosis.
Liestøl 2007 (23)	Case control	APS patients (n=52)<br>o APS patients on warfarin treatment (n=34)<br>o APS patients not on warfarin treatment (n=18)<br>o Thrombotic manifestations (n=37)<br>o Obstetric manifestations (n=8)<br>o Obstretic and thrombotic manifestations (n=7)	Age = N/S<br>Sex = N/S<br><br>Age = N/S<br>Sex = N/S	Patients positive for LA (without APS not on warfarin treatment) (n=29)<br>Control patients on warfarin treatment (n=38)<br><br>Healthy controls (n=53)	Age = N/S<br>Sex = N/S<br><br>Age = N/S<br>Sex = N/S<br><br>Age = 40.0 ± N/S<br>Sex = 37%	PPP (mixed 1:1 with PNP)<br>5.0 pM TF<br>(± APC 5 nM f.c.)<br>4 µM phospholipids	APC resistance (normalised)<br><br>ETP (normalised)	Normalised ETP (Median (95% CI)):<br>o APS patients warfarin: 0.63 nM/min (0.59-0.66)<br>o APS patients no warfarin: 0.94 nM/min (0.83-1.00)<br>o LA positive controls: 0.85 nM/min (0.83-0.93)<br>o Warfarin control patients: 0.73 nM/min (0.70-0.77)<br>o Healthy controls: 1.01 nM/min (0.96-1.05)<br><br>Normalised percent inhibition of ETP by APC (Median (95% CI)):<br>o APS patients warfarin: 33.8% (28.8-55.7)<br>o APS patients no warfarin: 52.0% (41.0-81.2)<br>o LA positive controls: 78.8% (73.9-95.8)<br>o Warfarin control patients: 114.9% (111.0-121.6)<br>o Healthy controls: 106.7% (105.7-107.5)	In APS patients and LA positive patients the mean normalised ETP was significantly lower compared to controls, this effect was independent of warfarin use.<br><br>The inhibitory effect of APC is significantly decreased in the APS patient group and the LA positive patient group compared to the control group. In addition, the ETP inhibition by APC was also significantly lower in APS patients compared to the LA positive control group, suggesting that APC resistance APS patients could be more clinically relevant compared to LA positive controls.
Matsumoto 2017 (24)	Case control	LA positive APS patients with thrombotic phenotype (n=10)	Age = N/S<br>Sex = N/S	LA positive controls (without APS) (n=10)<br><br>Healthy controls (n=20)	Age = N/S<br>Sex = N/S<br><br>Age = N/S<br>Sex = N/S	PPP<br>0.5 pM TF/0.3 µM Ellagic acid<br>(1.0 pM TF without Ellagic acid for APC resistance assessment)<br>(± APC 20 nM f.c.)<br>4 µM phospholipids	APC resistance (normalised)<br><br>Lag time, peak height (absolute)	TG parameters (mean ± SD):<br>o APS patients: Lag time (28.8 ± 11.8 min), Peak height (158 ± 99 nM)<br>o LA positive controls: Lag time (12.5 ± 7.7 min), Peak height (158 ± 75 nM)<br>o Healthy controls: Lag time (4.5 ± 0.3 min), Peak height (362 ± 23 nM)<br><br>Normalised PH inhibition by APC (mean ± SD):<br>o APS patients: 5±7%<br>o LA positive controls: 42±39%	CAT-TG cannot distinguish LA positive APS patients from LA positive controls.<br><br>LA positive APS patients and LA positive controls have a significantly increased resistance to APC compared to controls. In addition, APC resistance was significantly increased in the LA positive APS patient group compared to LA positive control group, suggesting that APC resistance in LA positive APS patients could be more clinically relevant compared to LA positive controls.
Ramirez 2021 (25)	Cross-sectional	Secondary APS patients (SLE) with thrombotic history (n=41)(TG only on n=24)	Age = 52.4 ± N/S<br>Sex = 19%	SLE patients (with aPL without thrombosis) (n=41)(TG only on n=25)<br><br>SLE patients (with thrombosis without aPL) (n=31)(TG only on n=14)<br><br>SLE patients (without aPL and thrombosis)	Age = 49.2 ± N/S<br>Sex = 14%<br><br>Age = 54.1 ± N/S<br>Sex = 19%<br><br>Age = 42.6 ± N/S<br>Sex = 19%<br><br>Age = N/S	PPP (mixed 1:1 with PNP in anticoagulated patients)<br>5.0 pM TF<br>(± APC and Protac)<br>4 µM phospholipids (PPP reagents Stago)	APC resistance (absolute)	Fifty-nine percent of all patients had APC resistance to either APC or Protac, with 29% exhibiting dual resistance.<br>Prevalence of APC resistance was homogeneous among all patient groups for both APC and Protac.	No significant difference in the resistance to APC or Protac was found between secondary APS patients and SLE patients controls.

				(n=43)(TG only on n=37)<br><br>Healthy controls (n=100)	Sex = N/S				
Zuily 2012 (26)	Case control	Primary APS patients (n= 38)<br><br>Secondary APS patients (SLE) (n=10)	Age = N/S<br>Sex = N/S<br><br>Age = N/S<br>Sex = N/S	SLE patients (without aPL) (n=13)<br><br>aPL carriers with SLE (without APS)(n=6)<br><br>aPL carriers (without APS and SLE) (n=24)<br><br>Controls (not further specified) (n=39)	Age = N/S<br>Sex = N/S<br><br>Age = N/S<br>Sex = N/S<br><br>Age = N/S<br>Sex = N/S<br><br>Age = N/S<br>Sex = N/S	PRP (platelet count adjusted to 150 x 10^9 platelets/L)<br>0.5 pM TF<br>(± APC 6.7 nM, 13.9 nM, 25 nM and 65 nM f.c.)	APC resistance (absolute)	IC_{50}-APC (the APC concentration that produces a 50% inhibition of ETP; median (IQR))<br>○ Primary APS: 15.3 (9.7-34.0)<br>○ Secondary APS: 64.1 (25.9-65.0)<br>○ SLE patients (without aPL): 27.3 (23.5-43.5)<br>○ Controls: 10.4 (8.5-15.8)<br>○ No difference in IC_{50}-APC between aPL carriers with or without SLE	Patients with APS have a significantly increased resistance to APC and this is even more remarked in patients with SLE.

Abbreviations: **aβ2GPI**, anti-Beta 2 glycoprotein I antibodies; **aDI**, anti-domain I antibodies; **AID**, autoimmune disease; **APC**, activated protein C; **aPL**, antiphospholipid antibodies; **APS**, antiphospholipid syndrome; **CAT**, calibrated automated thrombogram; **CI**, confidence interval; **ETP**, endogenous thrombin potential; **IC**, inhibitory concentration; **IEM**, inverted erythrocyte membranes; **IQR**, interquartile range; **LA**, lupus anticoagulants; **NPV**, negative predictive value; **N/S**, not specified; **OR**, odds ratio; **PNP**, pooled normal plasma; **PPP**, platelet poor plasma; **PPV**, positive predictive value; **PRP**, platelet rich plasma; **SD**, standard deviation; **SEM**, standard error of the mean; **SLE**, systemic lupus erythematosus; **TF**, tissue factor; **TG**, thrombin generation; **TM**, thrombomodulin; **VKA**, vitamin K antagonist; **VTE**, venous thromboembolism.

Supplemental Table 5: Quality assessment based on the Newcastle Ottawa Scale.

		Cross-sectional studies							
		**Selection** (max. 3)			**Comparability** (max. 2)		**Outcome** (max. 2)		**Stars** (*n*)
		**Represen-tativeness**	**Non-respondents**	**Ascertain-ment**	**Controls for factor 1**	**Controls for factor 2**	**Assessment**	**Statistical test**	
**Arachchillage**	2014		*	*			*	*	4
**Efthymiou**	2021		*	*			*	*	4
**Foret**	2021		*	*			*	*	4
**Ramirez**	2020			*			*	*	3

		Case control studies									
		**Selection** (max. 4)				**Comparability** (max. 2)		**Exposure** (max. 3)			**Stars** (*n*)
		**Case definition**	**Represen-tativeness**	**Selection of controls**	**Definition of controls**	**Controls for factor 1**	**Controls for factor 2**	**Ascertain-ment**	**Same method**	**Non-response rate**	
**Billoir**	2021	*			*			*	*	*	5
**Bloemen**	2016				*			*	*		3
**De Laat-Kremers**	2021	*		*	*			*	*	*	6
**Devreese**	2010	*		*	*			*	*	*	6
**Green**	2012	*			*			*	*	*	5
**Hanly**	2000			*	*			*	*	*	5
**Kremers**	2018	*			*	*	*	*	*	*	7
**Liestol**	2007	*		*	*			*	*	*	6
**Matsumoto**	2016	*			*			*	*	*	5
**Zuily**	2012		*		*			*	*	*	5

Uncategorized References

1. Boeer K, Cuznetov L, Loesche W. Thrombin generation as marker to estimate thrombosis risk in patients with abnormal test results in lupus anticoagulant routine diagnostics. Thrombosis Journal. 2013;11(1):1-7.
2. Brandt JT. Antibodies to β2-glycoprotein I inhibit phospholipid dependent coagulation reactions. Thrombosis and haemostasis. 1993;70(10):598-602.
3. Devreese K, Peerlinck K, Arnout J, Hoylaerts MF. Laboratory detection of the antiphospholipid syndrome via calibrated automated thrombography. Thrombosis and haemostasis. 2009;101(01):185-96.
4. Lean SY, Ellery P, Ivey L, Thom J, Oostryck R, Leahy M, et al. The effects of tissue factor pathway inhibitor and anti-b-2-glycoprotein-I IgG on thrombin generation. Haematologica. 2006;91(10):1360-6.
5. Lecompte T WD, Perret-Guillaume C, Hemker HC, Lacolley P, Regnault V. Hypercoagulability resulting from opposite effects of lupus anticoagulants is associated strongly with thrombotic risk. Haematologica. 2007(92(5)):714-5. .
6. Liestøl S, Sandset PM, Jacobsen EM, Mowinckel MC, Wisløff F. Decreased anticoagulant response to tissue factor pathway inhibitor type 1 in plasmas from patients with lupus anticoagulants. British journal of haematology. 2007;136(1):131-7.
7. Radin M, Barinotti A, Foddai SG, Cecchi I, Rubini E, Roccatello D, et al. Cerebrovascular events in patients with isolated anti-phosphatidyl-serine/prothrombin antibodies. Immunologic Research. 2021;69(4):372-7.
8. Regnault V, Béguin S, Wahl D, de Maistre E, Hemker HC, Lecompte T. Thrombinography shows acquired resistance to activated protein C in patients with lupus anticoagulants. Thrombosis and haemostasis. 2003;89(02):208-12.
9. Sheng Y, Hanly J, Reddel S, Kouts S, Guerin J, Koike T, et al. Detection of ‘antiphospholipid’antibodies: a single chromogenic assay of thrombin generation sensitively detects lupus anticoagulants, anticardiolipin antibodies, plus antibodies binding β 2-glycoprotein I and prothrombin. Clinical & Experimental Immunology. 2001;124(3):502-8.
10. Slavík L, Novak M, Ulehlova J, Prochazka M, Prochazkova J, Lattova V, et al. Possibility of coagulation system activation determination with tissue factor in pregnancy complications. Clin Lab. 2016;62(1851):6.
11. Zuily S, de Laat B, Guillemin F, Kelchtermans H, Magy-Bertrand N, Desmurs-Clavel H, et al. Anti–domain I β2-glycoprotein I antibodies and activated protein C resistance predict thrombosis in antiphospholipid syndrome: TAC (I) T Study. The journal of applied laboratory medicine. 2020;5(6):1242-52.
12. Zuily S, Regnault V, Guillemin F, Kaminsky P, Rat A-C, Lecompte T, et al. Superficial vein thrombosis, thrombin generation and activated protein C resistance as predictors of thromboembolic events in lupus and antiphospholipid patients. A prospective cohort study. Thrombosis research. 2013;132(1):e1-e7.
13. Arachchillage D, Efthymiou M, Mackie I, Lawrie A, Machin S, Cohen H. Anti-protein C antibodies are associated with resistance to endogenous protein C activation and a severe thrombotic phenotype in antiphospholipid syndrome. Journal of Thrombosis and Haemostasis. 2014;12(11):1801-9.
14. Billoir P, Miranda S, Levesque H, Benhamou Y, Le Cam Duchez V. Hypercoagulability Evaluation in Antiphospholipid Syndrome without Anticoagulation Treatment with Thrombin Generation Assay: A Preliminary Study. Journal of Clinical Medicine. 2021;10(12):2728.
15. Bloemen S, Wu XX, Devreese KM, de Laat B, Rand JH, Vasovic LV. Inverted erythrocyte membranes demonstrate β2GPI-antiphospholipid antibody interactions and membrane crosslinking. Thrombosis Research. 2016;146:89-94.
16. de Laat-Kremers RM, Wahl D, Zuily S, Ninivaggi M, Chayouâ W, Regnault V, et al. Deciphered coagulation profile to diagnose the antiphospholipid syndrome using artificial intelligence. Thrombosis Research. 2021;203:142-51.
17. Devreese K, Peerlinck K, Hoylaerts MF. Thrombotic risk assessment in the antiphospholipid syndrome requires more than the quantification of lupus anticoagulants. Blood, The Journal of the American Society of Hematology. 2010;115(4):870-8.
18. Efthymiou M, Lane PJ, Isenberg D, Cohen H, Mackie IJ. Comparison of Acquired Activated Protein C Resistance, Using the CAT and ST-Genesia® Analysers and Three Thrombin Generation Methods, in APS and SLE Patients. Journal of clinical medicine. 2022;11(1):69.

19. Foret T, Dufrost V, Salomon du Mont L, Costa P, Lakomy C, Lagrange J, et al. A new pro-thrombotic mechanism of neutrophil extracellular traps in antiphospholipid syndrome: impact on activated protein C resistance. Rheumatology. 2022;61(7):2993-8.
20. Green L, Safa O, Machin SJ, Mackie IJ, Ryland K, Cohen H, et al. Development and application of an automated chromogenic thrombin generation assay that is sensitive to defects in the protein C pathway. Thrombosis research. 2012;130(5):780-4.
21. Hanly JG, Smith SA. Anti-beta2-glycoprotein I autoantibodies, in vitro thrombin generation, and the antiphospholipid syndrome. The Journal of Rheumatology. 2000;27(9):2152-9.
22. Kremers RM, Zuily S, Kelchtermans H, Peters TC, Bloemen S, Regnault V, et al. Prothrombin conversion is accelerated in the antiphospholipid syndrome and insensitive to thrombomodulin. Blood advances. 2018;2(11):1315-24.
23. Liestøl S, Sandset P, MOWINCKEL MC, Wisløff F. Activated protein C resistance determined with a thrombin generation-based test is associated with thrombotic events in patients with lupus anticoagulants. Journal of Thrombosis and Haemostasis. 2007;5(11):2204-11.
24. Matsumoto T, Nogami K, Shima M. A combined approach using global coagulation assays quickly differentiates coagulation disorders with prolonged aPTT and low levels of FVIII activity. International journal of hematology. 2017;105(2):174-83.
25. Ramirez GA, Mackie I, Nallamilli S, Pires T, Moll R, Pericleous C, et al. Anti-protein C antibodies and acquired protein C resistance in SLE: novel markers for thromboembolic events and disease activity? Rheumatology. 2021;60(3):1376-86.
26. Zuily S, Aissa KA, Membre A, Regnault V, Lecompte T, Wahl D. Thrombin generation in antiphospholipid syndrome. Lupus. 2012;21(7):758-60.
